# Acute inhibition of the CNS-specific kinase TTBK1 significantly lowers tau phosphorylation at several disease relevant sites

**DOI:** 10.1371/journal.pone.0228771

**Published:** 2020-04-07

**Authors:** Gregory M. Dillon, Jaclyn L. Henderson, Channa Bao, John A. Joyce, Michael Calhoun, Brenda Amaral, Kristopher W. King, Bekim Bajrami, Dania Rabah

**Affiliations:** Biogen, Cambridge, MA, United States of America; McGill University, CANADA

## Abstract

Hyperphosphorylated tau protein is a pathological hallmark of numerous neurodegenerative diseases and the level of tau pathology is correlated with the degree of cognitive impairment. Tau hyper-phosphorylation is thought to be an early initiating event in the cascade leading to tau toxicity and neuronal death. Inhibition of tau phosphorylation therefore represents an attractive therapeutic strategy. However, the widespread expression of most kinases and promiscuity of their substrates, along with poor selectivity of most kinase inhibitors, have resulted in systemic toxicities that have limited the advancement of tau kinase inhibitors into the clinic. We therefore focused on the CNS-specific tau kinase, TTBK1, and investigated whether selective inhibition of this kinase could represent a viable approach to targeting tau phosphorylation in disease. In the current study, we demonstrate that TTBK1 regulates tau phosphorylation using overexpression or knockdown of this kinase in heterologous cells and primary neurons. Importantly, we find that TTBK1-specific phosphorylation of tau leads to a loss of normal protein function including a decrease in tau-tubulin binding and deficits in tubulin polymerization. We then describe the use of a novel, selective small molecule antagonist, BIIB-TTBK1i, to study the acute effects of TTBK1 inhibition on tau phosphorylation *in vivo*. We demonstrate substantial lowering of tau phosphorylation at multiple sites implicated in disease, suggesting that TTBK1 inhibitors may represent an exciting new approach in the search for neurodegenerative disease therapies.

**Significance statement**

The results described here are of significance because they represent the first demonstration, to our knowledge, that acute inhibition of TTBK1 with a small molecule inhibitor can lead to significant reductions in tau phosphorylation *in vivo*. In addition, our data implicates TTBK1 as the major kinase responsible for the phosphorylation of tau at S422 in the brain, a modification almost completely absent from normal adults but significantly elevated across a multitude of neurodegenerative diseases. Therefore, we believe that due to the CNS restricted expression of TTBK1, pharmacological inhibition of this kinase represents a promising therapeutic approach in the treatment of tauopathies.

## Introduction

Tau is a neuron-specific, microtubule-associated-protein (MAP) that plays a key role in regulating tubulin polymerization. Tau-tubulin binding arises from ionic interactions between basic and acidic regions on the tau and tubulin molecules, respectively [1,2]. This interaction is especially sensitive to post-translational modifications and it is well established that the degree of tau phosphorylation correlates with the ability of this protein to bind to microtubules and subsequently stabilize the cytoskeleton [3]. Normally the tau protein is enriched in neuronal axons; however, the phosphorylation state of tau is also critical to its intracellular sorting and propensity for aggregation [4,5]. Hyper-phosphorylated tau tangles are a pathological hallmark of many neurodegenerative diseases grouped as tauopathies [6,7]. In Alzheimer’s patients, the severity of tau pathology, and not amyloid-β, positively correlates with disease progression, cortical atrophy, and cognitive dysfunction [8,9]. Interestingly, recent data have highlighted that hyper-phosphorylated tau leads to several toxic gain of function events that are independent of tau aggregation, including effects on DNA integrity [10], mRNA translational selectivity [11], and synaptic vesicle trafficking [12]. In summary, this data indicates that regardless of the proposed pathological role of tau in disease, the hyper-phosphorylation of tau represents an early event in this disease-associated cascade.

Identifying kinases involved in the pathological phosphorylation of tau may lead to promising clinical targets. However, the ubiquitous tissue expression of most kinases and the promiscuity of their substrates represent a hurdle for drug discovery. Tau-tubulin kinase (TTBK) is a dual serine/threonine and tyrosine kinase originally identified in a screen for proteins enriched in the human brain [13]. TTBK has two isoforms, TTBK1 (1321a.a.) and TTBK2 (1244a.a.), that include a highly homologous catalytic domain (88% identity and 96% similarity) and a divergent C-terminal domain (43% identity and 58% similarity; Ikezu and Ikezu [14]). While TTBK2 is ubiquitously expressed across multiple tissues [15], the TTBK1 isoform is restricted to the brain, spinal cord, and testis [13]. Interestingly, little is known about the substrates of TTBK isoforms, but previous experiments using a combination of biochemical and cell-based assays have demonstrated the ability of these kinases to phosphorylate tubulin [16], tau [13], TDP-43 [17] and SV2A [18].

Accumulating evidence has linked the expression or activity of the tau kinase TTBK1 with neurodegenerative diseases. (1) Two independent studies in small cohorts of Spanish and Han-Chinese populations have identified multiple single nucleotide polymorphisms (SNPs) in the TTBK1 gene that are proposed to reduce protein expression and are associated with a decreased risk of late onset Alzheimer’s disease [19,20]. (2) TTBK1 can directly phosphorylate both tau [13] and TDP-43 [17] at phosphorylation sites enriched in the post-mortem tissue of AD (S422) and ALS (S409/S410) patients respectively (3) Using immunohistochemistry, TTBK1 protein has been shown to co-localize with both diffuse phosphorylated S422 tau in Alzheimer’s disease neurons [21] and immune-positive AT180 (Thr231) neurons in sections from the hippocampus of FTLD-tau patients [22]. (4) Increased TTBK1 protein expression has been observed in the brains of patients with Alzheimer’s disease [23], Frontotemporal lobar degeneration (FTLD)-tau, and FTLD-TDP43 [17,22]. (5) Finally, transgenic mouse lines overexpressing TTBK1 exhibit significant age-dependent memory impairments which are TTBK1 transgene dose-dependent [23,24]. Interestingly, an increase in neurodegeneration has been observed following TTBK1 overexpression in a variety of model systems including mice [25], *C*. *elegans* [22], and *Drosophila melanogaster* [26]. Therefore, the cumulative evidence linking TTBK1 to disease and the restriction of TTBK1 expression to the CNS makes TTBK1 an interesting target for the treatment of tauopathies.

In the current studies, we set out to determine whether acute inhibition of TTBK1 could represent a viable strategy for lowering tau phosphorylation in disease. First, we demonstrate in both HEK293 cells and primary neuron cultures that the overexpression or knockdown of TTBK1 regulates the phosphorylation of tau at disease relevant sites. Importantly, we show that the TTBK1-specific phosphorylation of tau leads to a decrease in tau-tubulin binding and subsequent deficits in tubulin polymerization. We demonstrate that acute treatment with a newly identified TTBK1 inhibitor, BIIB-TTBK1i, results in a dose dependent decrease in the phosphorylation of tau at several different sites in mice. By using chemical proteomics, we were able to show both TTBK1 target engagement and the exquisite kinome selectivity of BIIB-TTBK1i *in vivo*. These results represent the first demonstration that acute TTBK1 inhibition is a viable therapeutic approach for lowering tau phosphorylation.

## Results

### TTBK1 overexpression leads to direct and indirect phosphorylation of Tau at disease-relevant sites

**Fig 1:**
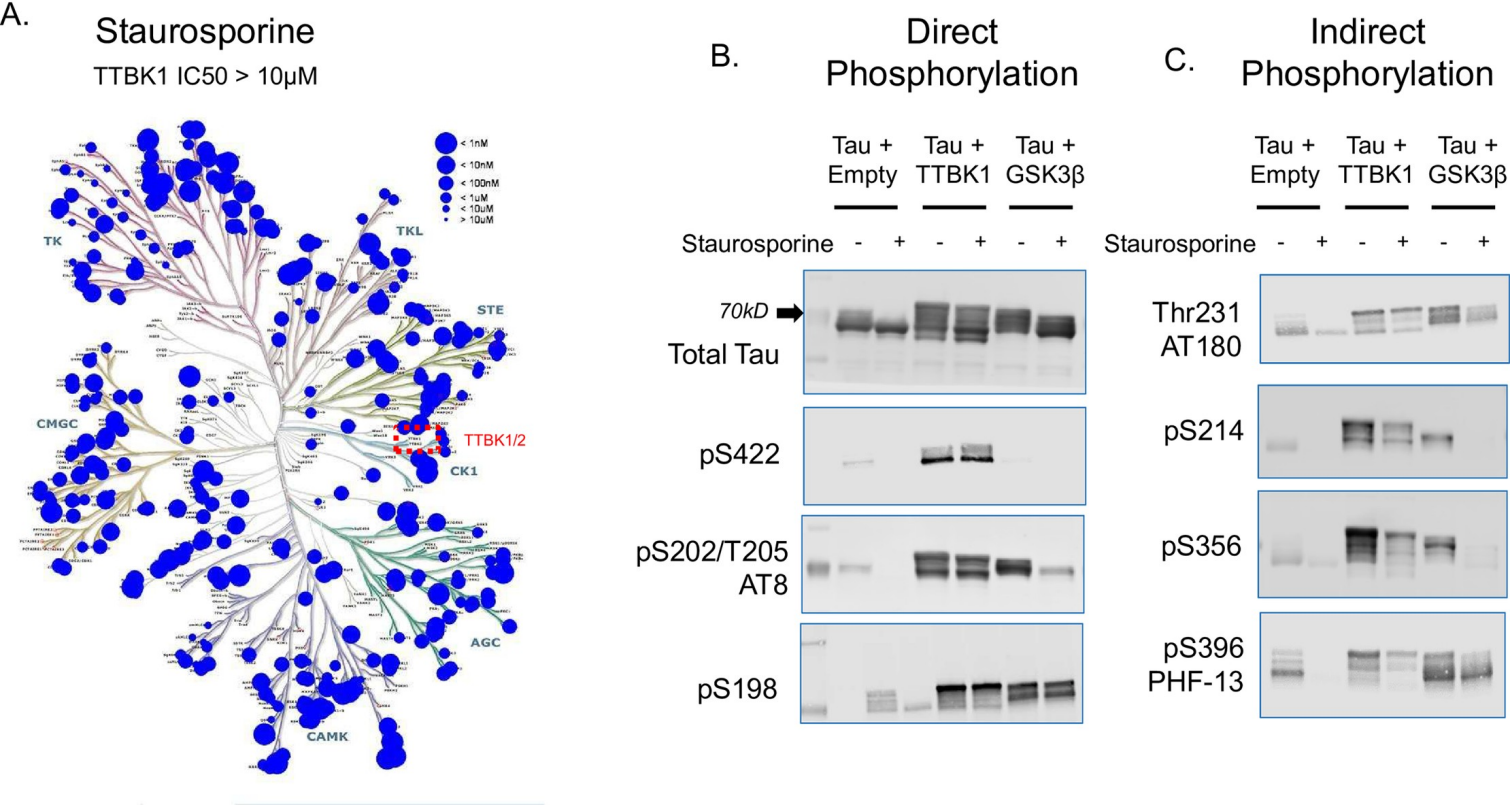
TTBK1 overexpression leads to direct and indirect phosphorylation of Tau at disease-relevant sites. (A) Phylogenetic tree of the human kinome illustrating the biochemical IC_50_ for Staurosporine against various kinases (data compiled from Davis et al., 2011 in addition to internal data on the affinity of Staurosporine for TTBK1/TTBK2). In HEK293 cells, plasmids expressing either full-length TTBK1 or the known tau kinase GSK3β were co-transfected with human Tau (2N4R) plus or minus Staurosporine (10μM) for one hour. TTBK1 overexpression caused a dramatic increase in the overall level of tau phosphorylation. (B-C) Representative western blots demonstrating sites of tau phosphorylation following TTBK1 overexpression that were either not affected by Staurosporine treatment (B) or those that were attenuated by Staurosporine (C).

Previous work using phospho-peptide mapping indicates that TTBK1 can phosphorylate tau at serine residues 198, 199, 202, 422 and at tyrosine 197; all sites enriched in paired helical filaments which characterize tauopathies [27–30]. In addition, TTBK1 has also been shown to colocalize with the immune-positive AT180 (Thr231) neurons in sections from the hippocampus of FTLD-tau patients [22]. However, it is unclear whether TTBK1 directly phosphorylates tau at these sites or whether it enhances the phosphorylation of tau through the activation of other kinases. To address this question, we took advantage of staurosporine, a pan kinase inhibitor, which inhibits most of the known tau kinases at low nanomolar concentrations (Fig 1A and S1 Table) but has an extremely low affinity for TTBK1 (>30uM IC_50_; Fig 1A). Plasmids expressing full-length TTBK1 or the known tau kinase GSK3β were co-transfected with human Tau (2N4R) in HEK293 cells plus or minus staurosporine at 10μM. At this concentration staurosporine will inhibit most of the endogenous kinases present in HEK293 cells but will not directly bind to the TTBK active site. TTBK1 overexpression led to the increase in tau phosphorylation at several sites; some previously identified including S422, AT8 (S202/S205) and S198 as well as additional novel sites like AT180 (Thr231), PHF-13 (S396), S214, and S356 (Fig 1B and 1C). At several of these sites (S422, S198, and AT8), the increase in tau phosphorylation following TTBK1 overexpression was not inhibited by staurosporine treatment, suggesting that TTBK1 is directly responsible for phosphorylation at these epitopes (Fig 1B). In contrast, phosphorylation of tau at several additional sites (AT180, S214, S396 and S356) were reduced following treatment with staurosporine. This indicates that the phosphorylation of tau at these epitopes was most likely through the activation of other kinases as a consequence of TTBK1 overexpression and not through TTBK1 kinase activity (Fig 1B). These results map out the tau-phosphorylation fingerprint that results from TTBK1 overexpression in a cellular system.

### TTBK1-mediated Tau phosphorylation reduces its affinity to bind to microtubules and its tubulin polymerization activity

**Fig 2:**
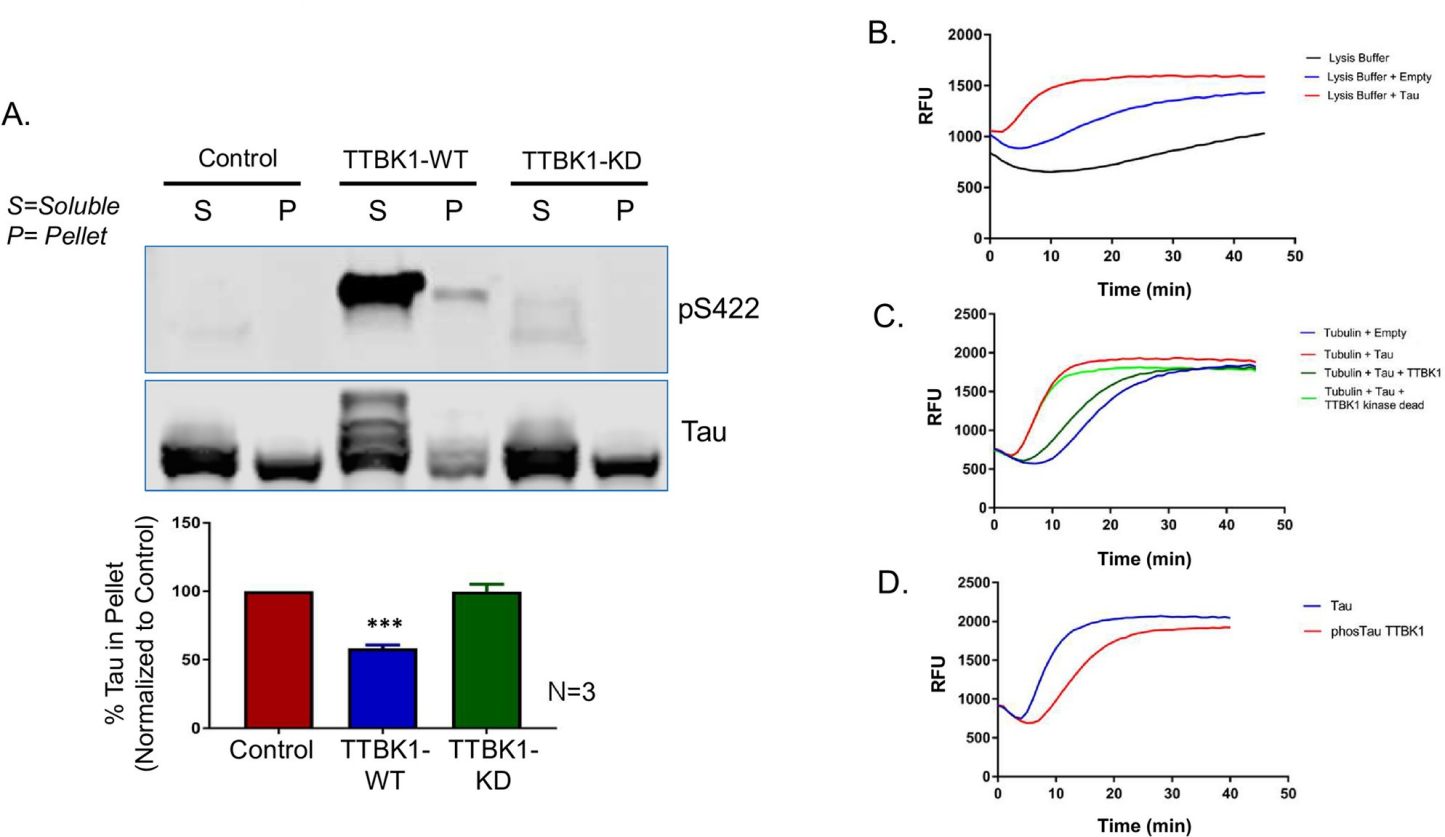
TTBK1-mediated phosphorylation reduces the affinity of Tau to bind microtubules and Tau’s tubulin polymerization activity. In HEK293T cells, plasmids expressing full-length TTBK1 or a kinase dead version of TTBK1 K63A were co-transfected with human Tau (2N4R). After 48 hours, cell lysates were subjected to ultracentrifugation to pellet both microtubules and microtubule associated proteins. (A) Top Panel: Representative western blot showing total tau found in both the soluble and pellet fraction. TTBK1 overexpression caused a significant decrease in the percentage of tau bound to microtubules in the pelleted fraction. Bottom Panel: Data was quantified based on western blot band intensity using the Licor and represented as means + standard error of the mean (SEM). Statistical significance was assessed using an unpaired t test (average of 3 experimental replicates; p<0.0001). There was no effect of the TTBK1 kinase dead plasmid on tau-tubulin binding. (B) Plasmids expressing either an empty vector control or human Tau (2N4R) were transfected into HEK293T cells. Lysates were added to a solution of recombinant porcine tubulin and the polymerization was measured using a fluorescence readout. The addition of human tau caused a left shift in the rate of tubulin polymerization when compared to HEK293 cells transfected with an empty plasmid. (C) The co-transfection of TTBK1 with human tau leads to a decrease in the rate of tubulin polymerization. This effect of TTBK1 on polymerization was kinase dependent. (D) To verify that the effect of TTBK1 on tubulin polymerization is tau dependent we performed the same assay using recombinant human tau protein alone or human tau co-expressed with TTBK1 in *E*. *coli* cells. Tubulin polymerization was slower with TTBK1 phosphorylated tau isolated from *E*. *coli* compared to tau alone.

Tau is a neuronal, microtubule associated protein that plays a key role during microtubule assembly [31]. Previously, it has been shown that the phosphorylation of tau reduces its affinity for microtubules and that this increase in soluble tau can lead to tau toxicity and aggregation [32–35]. To determine if TTBK1 phosphorylation of tau also regulates tau-microtubule binding, we co-transfected human tau into HEK293 cells with either full-length human TTBK1 or a kinase dead version of TTBK1 (TTBK1 K43A) and assessed the binding of tau to tubulin. Forty-eight hours following transfection, the cell lysates were first treated with Taxol (20μM) to stabilize existing tubulin polymers and then subjected to ultracentrifugation to pellet both the microtubules and microtubule-associated proteins (MAPs; protocol previously cited at Lansbergen et al., [36]). In HEK293 cells transfected with human tau, we found that tau protein was present in both the soluble (free in the cytosol) and pelleted fraction (bound to tubulin) (Fig 2A). When TTBK1 was co-expressed with tau, the percentage of tau in the pelleted fraction significantly decreased, demonstrating a reduction in the affinity of tau for microtubules as a result of TTBK1 phosphorylation (Fig 2A). Our previous data shows that TTBK1 directly phosphorylates tau at S422. Interestingly, we find that close to 100% of tau phosphorylated at S422 is present in the soluble fraction (Fig 2A, top panel of western blot). Our results also demonstrate that the effect of TTBK1 on tau-microtubule binding is phosphorylation dependent as co-transfection of tau with a kinase dead version of TTBK1 had no effect on the percentage of tau bound to microtubules (Fig 2A). Therefore, the phosphorylation of tau by TTBK1 reduces the ability of tau to bind to microtubules.

Since the binding of tau to microtubules is essential for promoting microtubule polymerization [37], we investigated the impact of TTBK1- mediated tau phosphorylation on the rate of tubulin polymerization. In this assay, lysates from HEK293 cells transfected with either human tau or a control plasmid were added to a solution of recombinant porcine tubulin. Tubulin polymerization was then measured using absorbance readings at 340 nm based upon the fact that light is scattered by microtubules at a rate proportional to the concentration of microtubule polymer [38]. Similar to previous findings [39], the addition of human tau significantly increased the rate of tubulin polymerization in our assay when compared to control transfected HEK293 cell lysates (Fig 2B). When TTBK1 was co-transfected with tau, it led to a significant reduction in tubulin polymerization, abolishing the previous enhancing effect of the addition of human tau (Fig 2C). This effect is kinase activity dependent as no shift in tubulin polymerization is seen following addition of the TTBK1 kinase dead plasmid (Fig 2C; S1 Fig). To verify that the effect of TTBK1 on tubulin polymerization is tau dependent, and not due to the phosphorylation of other microtubule-associated proteins present in mammalian cell lysates, we performed the same assay using recombinant human tau protein that was co-expressed with TTBK1 in E. coli cells (Signal Chem; tau-441, TTBK1-phosphorylated catalog #T08-50ON). In agreement with our previous experiments, these results conclusively demonstrate that tau phosphorylated by TTBK1 is significantly impaired in its ability to enhance tubulin polymerization (Fig 2D). Together, these data demonstrate that the phosphorylation of tau by TTBK1 reduces tau binding to microtubules thereby preventing the enhancement of tubulin polymerization by tau.

### TTBK1 knockdown reduces Tau phosphorylation in mouse primary neurons

**Fig 3:**
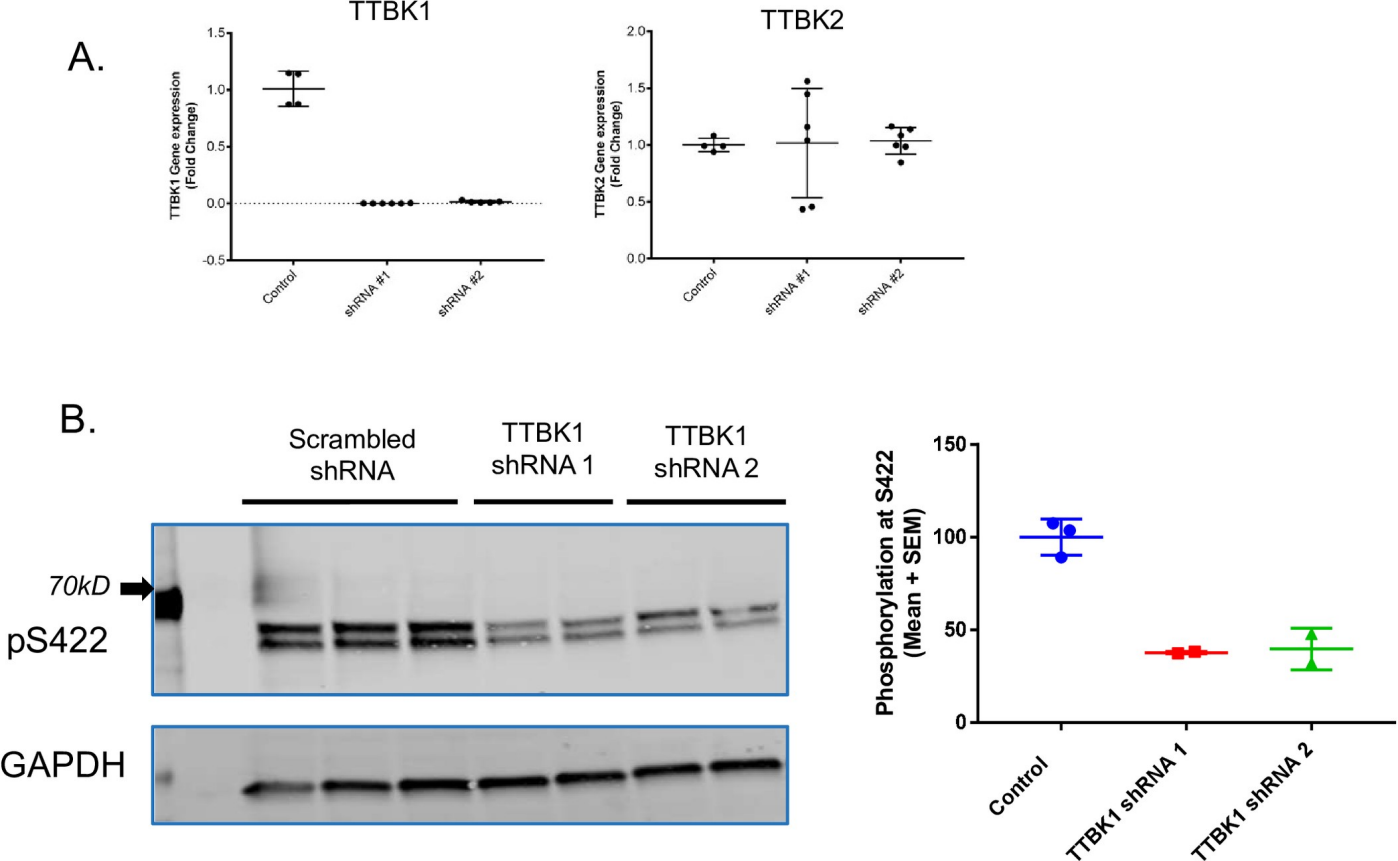
TTBK1 knockdown reduces Tau phosphorylation in mouse primary neurons. (A) Primary mouse neuron cultures were transduced with lentivirus expressing either scrambled or one of two specific TTBK1 shRNA sequences for seven days. Our data indicates that each of the TTBK1 shRNA sequences was efficient in completely knocking down the TTBK1 transcript with no consistent effect on the TTBK2 isoform (Each data point represents a biological replicate; n = 4 control, n = 6 shRNA sequence 1, and n = 6 shRNA sequence 2. Data presented as mean +/- SEM). (B) Left Panel: Western blot demonstrating reduction in the phosphorylation of tau at S422 following TTBK1 knockdown. Right panel: Quantification of Western blot using Licor (data represented as mean +/- standard error of the mean; each dot represents a biological replicate n = 3 control, n = 2 for each shRNA sequence).

The overexpression of tau can lead to an aberrant increase of tau in the soluble fraction resulting in tau mis-localization and phosphorylation patterns not present in healthy neurons. To investigate whether TTBK1 can phosphorylate endogenously expressed tau, we examined the effect of TTBK1 knockdown on tau phosphorylation in primary neuron cultures. Primary mouse neuron cultures were transduced with lentivirus expressing either a scrambled control or TTBK1-specific shRNA sequences. Our data indicates that seven days following transduction, each of the TTBK1 shRNA sequences (TTBK1 shRNA sequence 1: CTACTTCACCAAGCCCGATTA; TTBK1 shRNA sequence 2: ACATCAAGCCGTCCAACTTTG) was efficient in completely knocking down the TTBK1 transcript with no significant effect on the TTBK2 isoform (Fig 3A). To determine the effect of TTBK1 knockdown on tau phosphorylation, age-matched neuron cultures were treated with the Protein Phosphatase 2A(PP2A) inhibitor, okadaic acid (100 nM), to allow for tau phosphorylation by endogenous kinases. TTBK1 knockdown significantly reduced the amount of tau phosphorylation following okadaic acid treatment at S422 (Fig 3B), a site that we have previously shown to be directly phosphorylated by TTBK1 (Fig 1B).

**Fig 4:**
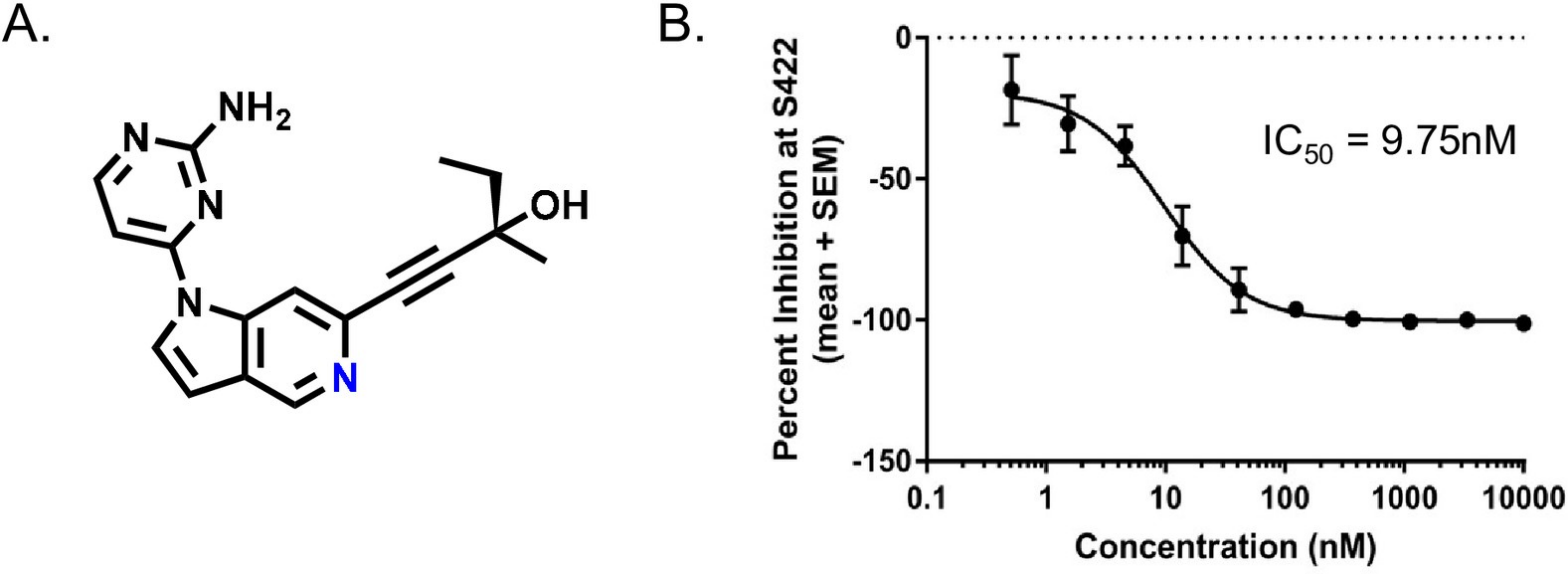
Identification of novel, small molecule TTBK1 inhibitor which dose dependently decreases tau phosphorylation at S422. (A) Structure of identified TTBK1 inhibitor–BIIB-TTBKi. (B) Full length TTBK1 and human tau (2N4R) were co-transfected in HEK293 cells. Phosphorylation of Tau at residue S422 was measured by FRET using the anti-Tau5 and anti-phospho S422 Tau antibodies conjugated with d2 (acceptor) and Tb cryptate (donor), respectively. BIIB-TTBK1i led to a dose dependent inhibition of phosphorylation at S422 with an average IC_50_ of 9.75 nM ± 1.38 (n = 5 experimental runs).

To verify that the effect of TTBK1 on tau phosphorylation is kinase dependent, we set out to identify small molecule inhibitors of TTBK1. After multiple rounds of optimization by the Biogen medicinal chemistry group, we identified a tool compound (herein named BIIB-TTBK1i; Fig 4A) that displayed potent inhibition of TTBK1-mediated tau phosphorylation in both biochemical and cell-based assays. Using a recombinant kinase assay, BIIB-TTBK1i inhibited TTBK1 kinase activity with an IC_50_ of ~9.5 nM in the presence of 10 μM ATP (S2B Fig). To measure the cellular potency of this compound, full length TTBK1 and human tau were co-transfected in HEK293 cells. In this assay, phosphorylation of tau at residue S422 was measured by FRET using the anti-Tau5 and anti-phospho S422 Tau (pS422) antibodies conjugated with d2 (acceptor) and Tb cryptate (donor), respectively (Cisbio Bioassays). BIIB-TTBK1i led to a dose dependent inhibition of phosphorylation at S422 with an average IC_50_ of 9.75 nM ± 1.38 (n = 5 experimental runs, Fig 4B). Of note, the addition of BIIB-TTBK1i in HEK293 cells at concentrations >500nM led to complete inhibition of tau phosphorylation at S422, implicating TTBK1 as the sole kinase responsible for phosphorylation at this epitope in our assay.

### Demonstration of *in vivo* target engagement and kinome selectivity of the novel small molecule BIIB-TTBKi

In order to profile the effects of TTBK1 inhibition on tau phosphorylation *in vivo*, we first determined a suitable dosing method for acute efficacy studies. We performed a pharmacokinetic experiment investigating the free drug levels of BIIB-TTBKi following intraperitoneal (I.P). or oral (P.O.) dosing at 35 mg/kg. Despite a rapid clearance rate in mouse, our data shows that we were able to achieve free drug concentrations of BIIB-TTBKi above the cellular IC_50_ for the first hour after dosing (S3 Fig).

**Fig 5:**
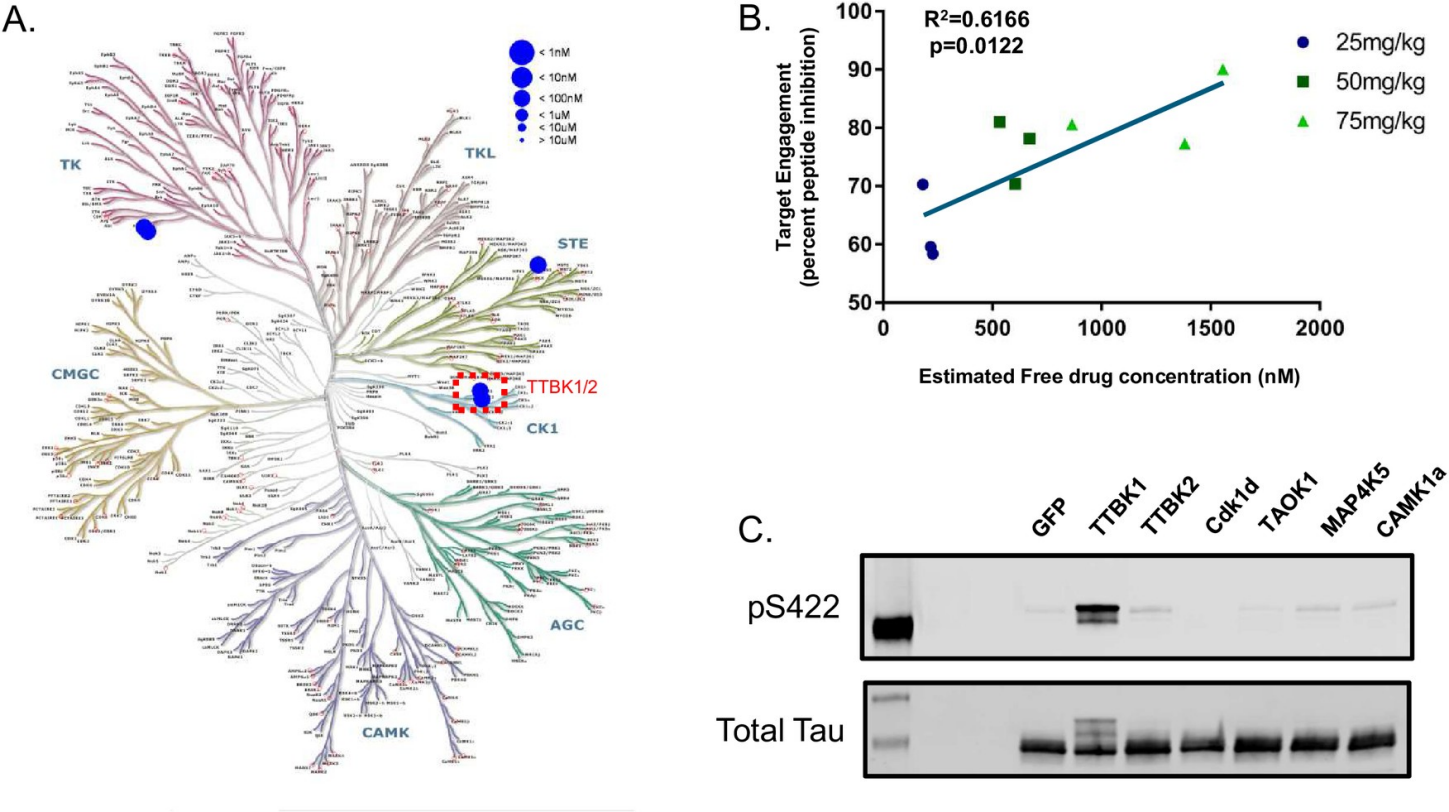
Demonstration of *in vivo* target engagement and kinome selectivity of the novel small molecule BIIB-TTBKi. BIIB-TTBK1i was administered I.P. at 25, 50, and 75 mg/kg in 2-month-old, male C57Bl/6 mice (n = 3 per treatment group). Mice were euthanized one hour following injection and homogenized brain lysates were labeled with Desthiobiotin-ADP-acyl phosphate probe (ADP probe). Desthiobiotinylated peptides were captured on streptavidin beads and peptides were quantified through LC-MS/MS parallel reaction monitoring method. Target engagement was calculated as the percentage of peptide signal lost when compared to the vehicle treated lysate. (A) Visualization of the off-target effects of BIIB-TTBKi at 75mg/kg. The ADP probe labelled around 150 unique kinases in mouse cortical brain lysates. Of the 150 kinases identified, BIIB-TTBKi (at 75mg/kg) demonstrated greater than 50% peptide inhibition at only three kinases apart from TTBK1/TTBK2. (B) A direct correlation was demonstrated between free levels of drug in the brain and target occupancy at the active site of TTBK1/TTBK2 (Target occupancy measured by competitive peptide inhibition with the small molecule; R^2^ = 0.6166; p = 0.0122).

To determine both *in vivo* target engagement and identify any potential off-target kinases inhibited by BIIB-TTBKi, we used a combination of desthiobiotin-ATP/ADP probes to covalently bind and biotinylate the conserved lysine residues at the active site of protein kinases (ActivX-KiNativ; Kyorin Pharmaceuticals, Ltd, Tokyo Japan). After dosing BIIB-TTBK1i at 25, 50, and 75 mg/kg IP in 2-month-old, male C57Bl/6 mice, mice were euthanized one hour following injection and homogenized brain lysates were labeled with Desthiobiotin-ADP-acyl phosphate probe (ADP probe). Lysates were trypsin digested and desthiobiotinylated peptides were captured on streptavidin beads. A list of 269 unique peptides were quantified through LC-MS/MS parallel reaction monitoring method. Target engagement was calculated as the percentage of peptide signal lost after addition of BIIB-TTBKi when compared to the vehicle treated lysate. Using the ADP probe, we were able to detect a total of 150 kinases in the mouse cortical brain lysates (Fig 5A). Of the 150 kinases detected, only 4 kinases (including TTBK1/TTBK2) were determined to have a target occupancy greater than 50% following treatment with BIIB-TTBK1i at 75 mg/kg (Fig 5A). Other than TTBK1, none of the identified off-target kinases have previously been shown to phosphorylate tau (see Table 1).

**Table 1:** List of kinases identified *in vivo* with greater than 50% occupancy one-hour following administration of our novel TTBK1 inhibitor.

Kinase	Sequence	Labeling Site	Percent Inhibition with BIIB-TTBKi at 75mpk
TTBK1, TTBK2	DIKPSNFAMGR	Lys2	82%
FES	LRADNTPVAVKSCR	Lys1	50.6%
PIP4K2C	TLVIKEVSSEDIADMHSNLSNYHQYIVK	ATP	86.3%
PIP5K3	GGKSGAAFYATEDDRFILK	ATP	90%

Target Engagement calculated as the percentage of ADP probe signal lost after addition of BIIB-TTBKi. One hour after BIIB-TTBKi at 75mg/kg was administered, only 4 kinases had greater than 50% reduction in signal.

These results indicate the high selectivity of BIIB-TTBK1i across the kinome. In addition, our data show a direct correlation in these samples between free drug levels and target occupancy at the active site of TTBK1/TTBK2 as measured by competitive peptide inhibition (Fig 5B). Together the use of chemical proteomics here allowed us to show both selectivity and dose dependent target coverage *in vivo* using our novel inhibitor.

To determine, if there were additional off-target kinases for which our ADP probe had low affinity, we added either the original ADP probe or an additional ATP-based probe to mouse cortical brain lysates in combination with BIIB-TTBK1i at 1μM *ex vivo*. The *ex vivo* addition of BIIB-TTBK1i at 1μM caused the inhibition of significantly more off-target kinases than were previously identified in the *in vivo* experiment (Table 2. total of 13 kinases measured to have greater than 50% occupancy).

**Table 2:** List of off-target kinases following the *ex vivo* addition of either the ATP and/or ADP probe.

Kinase	Sequence	Labeling Site	Probe	Percent Inhibition at 1μM
TTBK1, TTBK2	DIKPSNFAMGR	Lys2	ADP probe	95.8
CaMK1a	LVAIKCIAK	Lys1	ATP probe	69.2
CaMK1a	DLKPENLLYYSLDEDSK	Lys2	ATP probe	63.8
CaMK1a	LVAIKCIAK	Lys1	ADP probe	69.3
CaMK1a	DLKPENLLYYSLDEDSK	Lys2	ADP probe	64.7
CK1d, CK1e	DVKPDNFLMGLGKK	Lys2	ATP probe	55.5
FER	TPVAIKTCKEDLPQELK	Lys1	ATP probe	74.9
FER	QEDGGVYSSSGLKQIPIK	Protein Kinase Domain	ATP probe	69.4
FER	TPVAIKTCKEDLPQELK	Lys1	ADP probe	79.1
FER	QEDGGVYSSSGLKQIPIK	Activation Loop	ADP probe	66.7
FES	LRADNTPVAVKSCR	Lys1	ATP probe	61.2
FES	LRADNTPVAVKSCR	Lys1	ADP probe	79.8
MAP4K5	NVHTGELAAVKIIK	Lys1	ATP probe	58.7
MAP4K5	NVHTGELAAVKIIK	Lys1	ADP probe	69.7
MLK2	DLKSINILILEAIENHNLADTVLK	Lys2	ATP probe	60.1
PIP4K2C	TLVIKEVSSEDIADMHSNLSNYHQYIVK	ATP	ATP probe	76.5
PIP4K2C	VKELPTLKDMDFLNK	ATP	ATP probe	67.9
PIP4K2C	TLVIKEVSSEDIADMHSNLSNYHQYIVK	ATP	ADP probe	88.8
PIP5K3	GGKSGAAFYATEDDRFILK	ATP	ATP probe	95.9
PIP5K3	GGKSGAAFYATEDDRFILK	ATP	ADP probe	90.2
PITSLRE	DLKTSNLLLSHAGILK	Lys2	ATP probe	63.3
TAO1	TNEVVAIKK	Lys1	ADP probe	75.9
TAO1, TAO3	DIKAGNILLTEPGQVK	Lys2	ATP probe	69.5
TAO1, TAO3	DIKAGNILLTEPGQVK	Lys2	ADP probe	66.8
TAO2	DVKAGNILLSEPGLVK	Lys2	ATP probe	93.2
TAO2	NSEVVAIKK	Lys1	ADP probe	86.4
TAO2	DVKAGNILLSEPGLVK	Lys2	ADP probe	82.6

Target Engagement calculated as the percentage of ADP probe signal lost after addition of BIIB-TTBKi. BIIB-TTBKi was spiked into cortical lysate at final concentration of 1μM.

In particular, the ATP probe identified two unique kinases when compared to the ADP probe: CK1delta and PITSLRE (CDC2L1). To further solidify that any effects of BIIB-TTBK1i on tau phosphorylation were specific to TTBK1 inhibition, we co-transfected HEK293 cells with human tau plus any of the identified off-target kinases previously identified to phosphorylate tau (http://cnr.iop.kcl.ac.uk/hangerlab/tautable; Cdk1d, TAOK1, MAP4K5 and CAMK1a). We did not see an increase in tau phosphorylation at S422 after the overexpression of the potential off-target kinases of BIIB-TTBK1i (Fig 5C). Together, these results demonstrate the advantages of using chemical proteomics to measure *in vivo* target engagement as well as reinforce the specificity of our tool compound for TTBK1.

### Acute TTBK1 inhibition reduces tau phosphorylation at disease relevant sites *in vivo*

**Fig 6:**
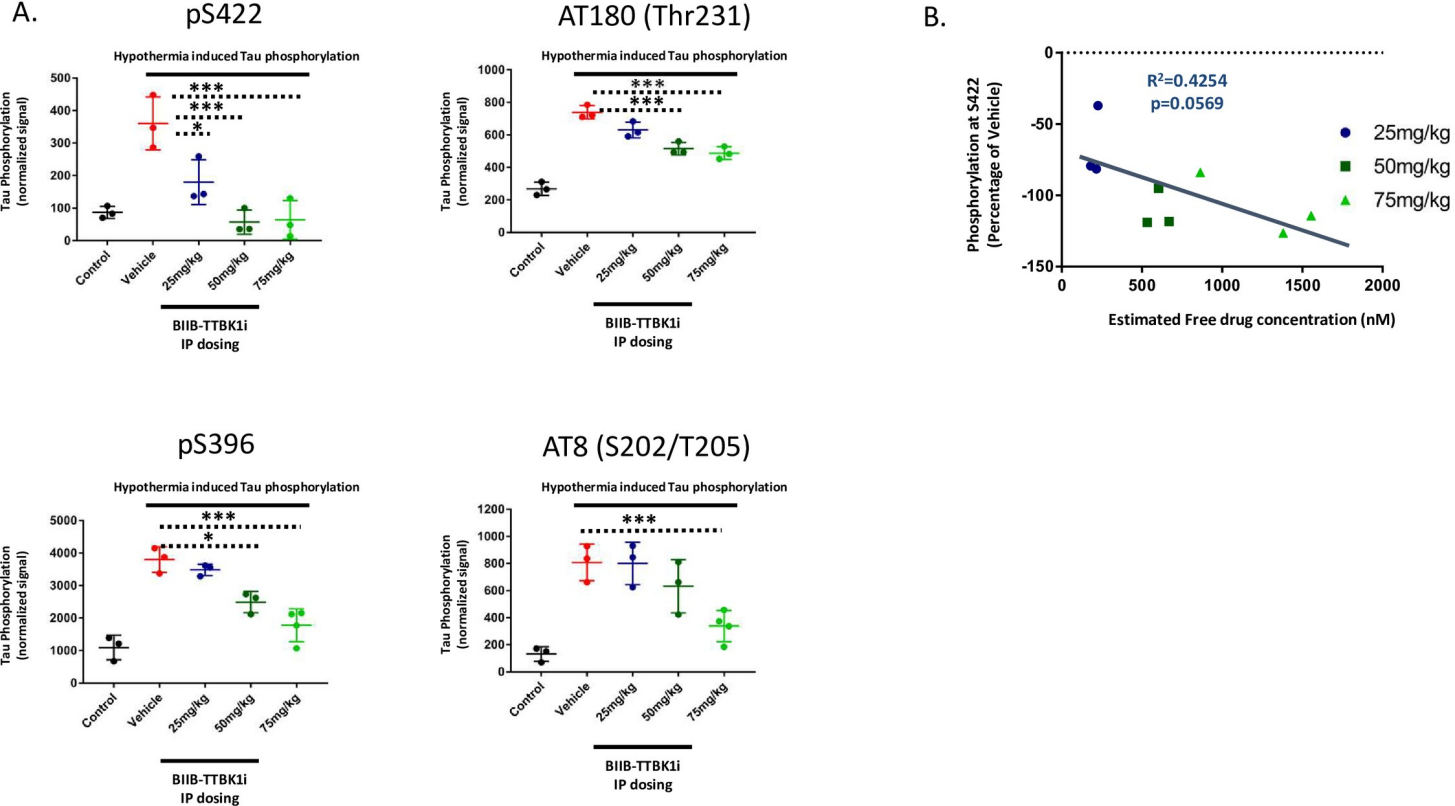
Acute TTBK1 inhibition reduces tau phosphorylation at disease relevant sites *in vivo*. We administered the TTBK1 inhibitor, BIIB-TTBK1i, at 25, 50, and 75mg/kg, five minutes prior to isoflurane-induced hypothermia in 2-month-old, male C57Bl/6 mice (n = 3 mice per dose group). (A) At the highest dose tested (75mg/kg), there was a significant decrease in tau phosphorylation at several sites when quantified by immunoblotting (S422, AT180, S396, and AT8). Phosphorylation signal was normalized to the level of total tau in each sample. Data is represented as the mean +/- SEM. For each phospho-epitope, treatment significance was determined first by one-way ANOVA between all groups; For each dose group, Tukey’s comparison was used to determine significance versus the Vehicle treated control. (B) Correlation within each brain sample between the level of tau phosphorylation at S422 and the measured concentration of free drug (R^2^ = 0.4254; p = 0.0569).

Under basal conditions, tau phosphorylation is tightly regulated by opposing kinase and phosphatase activities. In mice, isoflurane induced hypothermia causes a dramatic increase in tau phosphorylation through the temperature dependent inhibition of PP2A [40]. In the current experiment, we used isoflurane induced hypothermia in C57Bl/6 mice to increase tau phosphorylation in order to test the effects of TTBK1 inhibition. By using wild-type mice we ensured that both the levels of tau and tau kinases were at endogenous levels. We administered the TTBK1 inhibitor, BIIB-TTBK1i, at 25, 50, and 75mg/kg, I.P. five minutes prior to isoflurane-induced hypothermia in 2-month-old, male C57Bl/6 mice. Based on our *in vitro* potency assays and pharmacokinetic parameters, the highest dose used (75 mg/kg) was projected to achieve an IC_80_ of TTBK1 inhibition for the one hour of hypothermia treatment (S3 Fig). Similar to previous reports [40], our results show a dramatic increase in tau phosphorylation following hypothermia at each of the phospho-epitopes tested (Fig 6A). Acute treatment with our novel, TTBK1 inhibitor—BIIB-TTBK1i - caused a dose dependent decrease in the phosphorylation of tau at several different sites (For each phospho-epitope treatment significance was determined first by one-way ANOVA between all groups; For each dose of BIIB-TTBKi, Tukey’s multiple comparisons were used to determine significance versus the Vehicle treated control). Many of the phospho-epitopes significantly lowered after BIIB-TTBKi treatment were previously shown by our *in vitro* experiments to be direct and/or indirect sites of TTBK1 (Fig 6A). These results are the first demonstration that acute TTBK1 inhibition can lower tau phosphorylation *in vivo*. Notably, the levels of phosphorylation at S422 on tau were completely restored to basal levels one hour following treatment with BIIB-TTBK1i at either 50 or 75mg/kg (Compared to Vehicle: 25mg/kg p = 0.0213; 50mg/kg p = 0.0005; 75mg/kg p = 0.0007). This further confirms that S422, a site implicated in the early stages of neurofibrillary tangle formation in disease [41], is likely solely phosphorylated by TTBK1 in the brain. Of the phosphorylation sites profiled, S422 was found to be the most sensitive to acute TTBK1 inhibition but we also saw a significant decrease in tau phosphorylation at several other sites using antibodies for AT180 (Thr231; Compared to Vehicle: 25mg/kg p = 0.0571; 50mg/kg p = 0.0005; 75mg/kg p = 0.0002), AT8 (S202/T205; Compared to Vehicle: 25mg/kg p>0.999; 50mg/kg p = 0.5391; 75mg/kg p = 0.0066) and S396 (Compared to Vehicle: 25mg/kg p = 0.8492; 50mg/kg p = 0.011; 75mg/kg p = 0.0002).

Following isoflurane induced hypothermia, one cortical hemisphere was taken for western blot analysis and one cortical hemisphere was used to determine the levels of BIIB-TTBK1i in the brain. Our results demonstrate an inverse correlation between the amount of free drug detected in the brain and levels of tau phosphorylation at S422 (Fig 6B). Based on our data at the 25mg/kg dose we were able to show an average reduction in tau phosphorylation at S422 of 60% associated with a concentration of 207nM of free drug one hour after dosing. At the 50mg/kg dose, we see a complete reversal of the hypothermia induced tau phosphorylation at S422 with an average of 602nM of free drug in the brain. The dose responsive effects of BIIB-TTBK1i on tau phosphorylation lends further evidence to the specificity of our inhibitor towards TTBK1 directed tau phosphorylation. In conclusion, these results demonstrate that TTBK1 inhibition can lower tau phosphorylation at disease relevant sites *in vivo*.

## Discussion

The reduction of tau phosphorylation through the inhibition of tissue specific kinases is an attractive therapeutic approach for the treatment of Alzheimer’s disease and other tauopathies. The data presented here represent the first proof of biology that acute inhibition of TTBK1, a CNS specific tau kinase, can lower tau phosphorylation at disease relevant sites *in vivo*. Our results show that TTBK1 can phosphorylate tau both directly and indirectly leading to a reduction in the affinity of tau for microtubules thereby preventing the enhancement of tubulin polymerization by tau. The restriction of TTBK1 expression to the CNS and its ability to phosphorylate tau at S422, a phosphorylation site previously shown to be increased in a variety of neurodegenerative diseases, makes TTBK1 an exciting target for the treatment of tauopathies.

In a cell free system, numerous kinases have been reported to phosphorylate tau; however, these interactions may not reflect physiological kinase-substrate interactions which rely on factors such as cell compartment localization, kinase phosphorylation state, and substrate priming. Our results show that in HEK293 cells, co-expression of human full-length TTBK1 and human Tau (2N4R) leads to a significant increase in tau phosphorylation at numerous sites spanning the entire tau protein sequence. Making use of the fact that the pan kinase inhibitor staurosporine has almost no activity on TTBK1 catalytic activity (IC_50_ > 30μM) we were able to identify both direct and indirect phosphorylation sites of TTBK1 on tau. Our data are the first to identify several novel phosphorylation sites on tau that are either directly (S202/205; AT8) or indirectly (S214, Thr231, S396) regulated by TTBK1 activity. Importantly, we have confirmed in primary neuron cultures that TTBK1 knockdown can reduce tau phosphorylation levels at S422 when both tau and TTBK1 are present at endogenous levels. The indirect phosphorylation of tau which results from TTBK1 overexpression may occur through the activation of other kinases by TTBK1 or by the priming of tau through phosphorylation at other sites. Previously, Sato et al., [23] have shown an approximate 3-fold increase in CDK5 activity in TTBK1 overexpressing mice and many of the indirect phosphorylation sites on tau profiled here are known CDK5 epitopes (http://cnr.iop.kcl.ac.uk/hangerlab/tautable). Further work is needed to determine if TTBK1 overexpression in this cell system also initiates aberrant CDK5 activity driving indirect tau phosphorylation.

**Fig 7:**
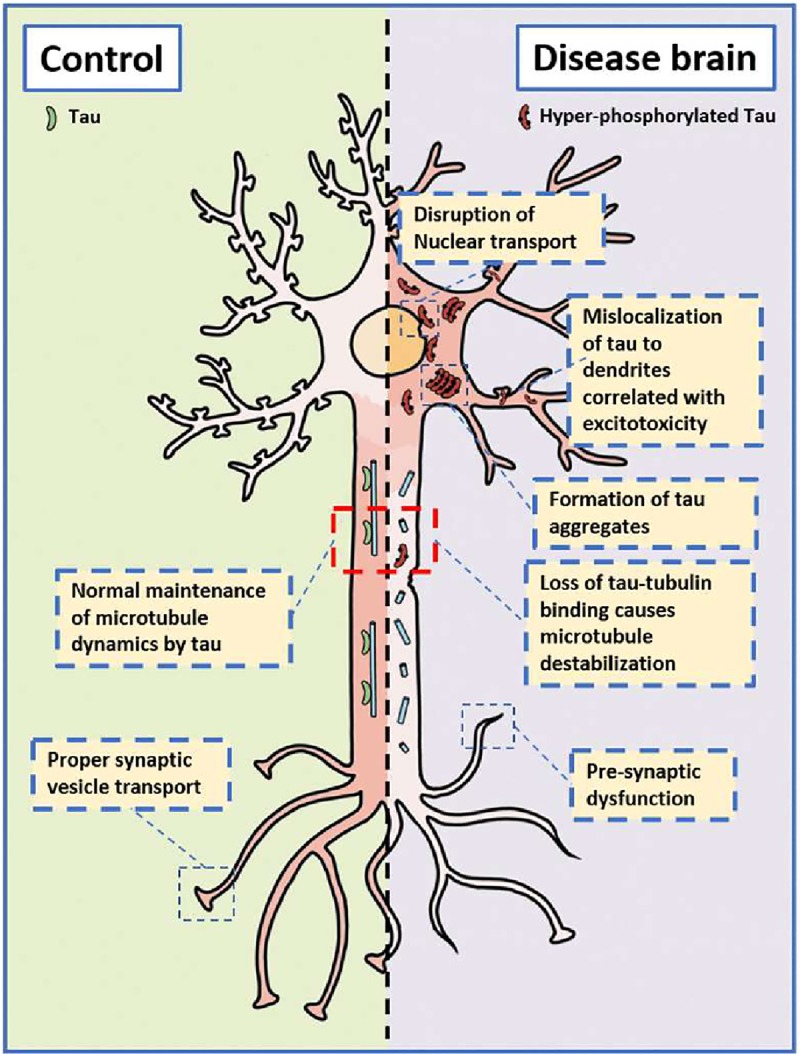
Model: Hyperphosphorylation of tau by TTBK1 can cause both a loss of normal tau function and a toxic gain of function as tau is mislocalized within the neuron.

The main physiological role of tau appears to be in the promotion of microtubule assembly [31] and previously this enhancement of tubulin polymerization has been shown to be dependent on Tau-tubulin binding [1,2]. Here we demonstrate that the phosphorylation of tau by TTBK1 reduces the binding of tau to microtubules resulting in a significant decrease in the rate at which tau promotes tubulin polymerization. Our experiments using purified recombinant proteins from *E*. *Coli* conclusively demonstrate that the effects of TTBK1 on tubulin polymerization are Tau dependent. Mutations in the tau gene (*MAPT*) are known to co-segregate with the disease of frontotemporal dementia with parkinsonism linked to chromosome 17 (FTDP-17). Preclinically, a number of *MAPT* mutations have been shown to effect tau-tubulin binding [42] and promote tau hyper-phosphorylation [43]. These observations provide direct genetic evidence indicating that decreasing the affinity of tau for microtubules can lead to tau pathology and dementia. Tau is predominantly found in neuronal axons however aberrant phosphorylation and subsequent mislocalization of tau protein to the nucleus or synapse can lead to a toxic gain of function. Several new lines of evidence show that aside from microtubule binding, the hyperphosphorylation of tau affects a variety of cellular processes including RNA translational selectivity [11], nuclear import/export [44], pre-synaptic vesicle motility [45] and post-synaptic excitotoxicity [46]. Therefore, one could speculate that the aberrant phosphorylation of tau by kinases (such as TTBK1) could begin a toxic cascade in neurons whereby hyperphosphorylated tau unhooks from the microtubule network, mislocalizes within the cell, and eventually develops its toxic gain of function (Fig 7).

The presence of intraneuronal tangles composed of hyperphosphorylated tau are a pathological hallmark of many neurodegenerative diseases grouped as tauopathies [6,7]. Many of the tau phosphorylation sites regulated by TTBK1, specifically S422, are found upregulated in a multitude of diseases including: AD, Down’s syndrome, FTDP-17, Progressive Supranuclear Palsy (PSP), Corticobasal Degeneration (CBD), Pick’s Disease, and Chronic traumatic encephalopathy (CTE; Bussiere et al., [47]; Kanaan et al., [48]; Pascual G et al. [49]). This implicates kinases which phosphorylate tau at S422 as potential instigators in the formation of neurofibrillary tangles common across tauopathies. We were interested in identifying small molecule inhibitors of TTBK1 as potential drug candidates and discovered BIIB-TTBK1i to have low nanomolar cellular potency and PK properties suitable for acute rodent studies. To demonstrate both *in vivo* target engagement and the off-target profile of BIIB-TTBK1i, we used the chemoproteomics platform—KiNativ [50,51]. This novel approach allowed us to show a direct correlation between the free drug levels of our small molecule BIIB-TTBKi and target occupancy at the TTBK1/TTBK2 active site in the brain. In addition, our results show that the ADP probe used here labelled around 150 unique kinases in mouse cortical brain lysates. Of the 150 kinases identified, BIIB-TTBKi (at the highest dose tested, 75mg/kg) demonstrated greater than 50% peptide inhibition at only three kinases apart from TTBK1/TTBK2. Therefore, this approach allowed us to show the exquisite selectivity of BIIB-TTBKi across the kinome while at the same time establishing an expected range of target occupancy in the tissue of interest.

Mouse models of Alzheimer’s disease commonly use the transgenic overexpression of genes involved in the production of amyloid β (APP, PSEN1/2) and/or Tau (MAPT) and many of these models have been shown to recapitulate the hyperphosphorylated tau tangles characteristic of disease [52,53]. However, in human tauopathies, disease progression is not associated with an overall increase in tau protein expression but rather an imbalance of Tau isoforms or unknown factors leading to tau hyper-phosphorylation. To avoid potential confounds from tau overexpression, we used isoflurane-induced hypothermia to induce tau phosphorylation in wild-type mice as a means to test the physiological role of TTBK1 on tau phosphorylation. In the hypothermia model, we administered BIIB-TTBKi at a maximum concentration of 75mg/kg, a dose we show here through chemical proteomics to only inhibit 3 additional kinases (none of which have previously been shown in the literature or in our lab–Figure—to phosphorylate tau at S422) in the mouse brain. Our results are the first demonstration that acute TTBK1 inhibition can significantly lower tau phosphorylation at several disease relevant sites including S422, S202/S205 (AT8), S396, and T231 (AT180). Hypothermia induces tau phosphorylation by inhibiting the activity of protein phosphatase 2A (PP2A; Planel et al. [40,54]) the major phosphatase of Tau in the brain [55–57]which accounts for more than 70% of the total tau-phosphatase activity [57]. Interestingly, numerous studies have described a reduction in both the expression and activity of PP2A in the brains of AD patients [57–62]. These results implicate hypothermia as an acute way to model the imbalance of kinase and phosphatase activity which may occur during aging or disease. It is important to note that in the current experiments–hypothermia was used as a quick way to test the pharmacodynamic effects of our small molecule kinase inhibitors after elevating the levels of tau phosphorylation in the brain. Future experiments will be needed to determine whether chronic inhibition of TTBK1, presumably over the course of several months, would ameliorate the cognitive deficits demonstrated in tau transgenic models of disease [63,64].

Previous experiments have indicated that TTBK1 protein expression is significantly elevated in brains of both AD [23] and FTLD-TDP/FTLD-tau patients [22]. Our internal data also shows a modest, but significant, increase in TTBK1 transcript when comparing post-mortem, late-stage AD tissue and age matched controls (Controls n = 11, AD n = 10, Average Braak Stage 6; S4 Fig). However, when using a targeted proteomics approach (total of 16 unique TTBK1 peptides normalized to GAPDH) to quantify total TTBK1 protein levels, we were unable to show an increase in TTBK1 protein expression at earlier stages of the disease (Control n = 10, AD n = 10, Average Braak Stage 3–4; S5 Fig). Regardless of whether TTBK1 expression or activity increases during disease progression, our current experimental model implies that tau hyperphosphorylation can be induced by basal TTBK kinase activity simply due to a shift in the kinase/phosphatase equilibrium. Therefore, it is possible that early in disease the inhibition of phosphatase activities could permit key kinases like TTBK1 to phosphorylate tau.

Together, our *in vitro* and *in vivo* data indicate that S422 is a direct phosphorylation site of TTBK1 on tau. Previous studies have shown that phosphorylation of tau at S422 is almost completely absent in cognitively normal adults and in the fetal brain [49,65]). In contrast, significant increases in the phosphorylation of tau at S422 are detected early in AD [66,67] and the number of tau-S422 immuno-positive neurons correlates with cholinergic neuron loss and cognitive impairment [41]. Intriguingly, several experiments have shown that antibodies directed at tau S422 can alleviate disease phenotypes in a variety of transgenic mouse models [68–70]. Very few kinases have been shown to phosphorylate tau at S422 [71,72] and in our *in vitro* and *in vivo* models, TTBK1 inhibition almost completely abolished phosphorylation at this site. Therefore, our data implicates TTBK1 as the major kinase responsible for the phosphorylation of tau at S422 in the brain.

In conclusion, although both the tau species and the cellular mechanisms that lead to tau toxicity are unknown, it is clear that tau pathology correlates with disease progression and that the hyper-phosphorylation of tau is an early event in this disease cascade. Our data demonstrates that TTBK1, a CNS specific kinase, phosphorylates tau at several sites upregulated in disease including S422. These results are the first demonstration that acute inhibition of TTBK1 with a small molecule *in vivo* can lead to significant reductions in tau phosphorylation. Therefore, we believe that due to the CNS restricted expression of TTBK1, pharmacological inhibition of this kinase represents a promising therapeutic approach in the treatment of tauopathies.

## Materials and methods

### Cell culture and transfection

HEK293 cells obtained from American Type Culture Collection (ATCC, Manassas, VA) were cultured in Dulbecco's modified Eagle's medium (DMEM) supplemented with 10% fetal bovine serum (FBS), 10 units/ml penicillin, and 10μg/ml streptomycin (all reagents were purchased from Gibco, Waltham, MA). Cell cultures were maintained in a humidified 5% (v/v) CO_2_/air environment at 37°C. When the cells reached 50–80% confluence, they were transfected with a complex consisting of a 3:1 ratio of FuGENE 6 Transfection Reagent (Promega, Madison, WI) and plasmid DNA. Plasmid DNA was balanced out across reactions using an empty plasmid so that the same amount of DNA was added per well. This transfection complex was prepared following the manufacturers protocol and added directly to the cell media for 24 or 48 hours prior to lysis.

### SDS-PAGE and western blot

Samples were lysed in Pierce RIPA lysis and extraction buffer (Thermo Fisher Scientific, Waltham, MA) supplemented with 1% Halt protease and phosphatase inhibitor cocktail (Thermo Fisher Scientific, Waltham, MA) and centrifuged at 14000 x g for 20 min at 4°C to clear the lysate. Protein concentrations were determined using the Direct Detect Infrared Spectrometer (EMD Millipore Corp., Burlington, MA) and 10μg of each sample was denatured in 6X SDS sample buffer (Boston Bioproducts, Ashland, MA) for 7 minutes at 90°C. Proteins were loaded into a Criterion 7.5% tris-glycine gel (Bio-Rad, Hercules, CA) and separated by SDS-PAGE at 120V for 120 minutes. The gel was transferred to an IBlot2 nitrocellulose membrane (Invitrogen, Carlsbad, CA), blocked with TBST blocking buffer (Li-cor Biosciences, Lincoln, NE) for 1 hour, and washed three times with TBST. The membrane was probed with primary antibodies (1:1000 dilution) in antibody dilution buffer (1:1 TBST blocking buffer and 1X TBST) overnight at 4°C. The following antibodies were used for immunoblotting: TTBK1 (Cat# PA5-20686, Invitrogen, Carlsbad, CA), Tau-5 (Cat# ab80579, Abcam, Cambridge, UK), phospho-Tau S422 (Cat# ab79415, Abcam, Cambridge, UK), S198 (Cat# ab79540, Abcam, Cambridge, UK), AT8 S202/T205 (Cat# MN1020, Invitrogen, Carlsbad, CA), S214 (Cat# ab170892, Abcam, Cambridge, UK), AT180 T231 (Cat# MN1040, Invitrogen, Carlsbad, CA), S262 (Cat# ab131354, Abcam, Cambridge, UK), S356 (Cat# ab75603, Abcam, Cambridge, UK), S396 (Cat# ab109390, Abcam, Cambridge, UK), GAPDH (Cat# 5174S, Cell Signaling Technology, Danvers, MA), and β-actin (Cat# 926–42212, Li-cor Biosciences, Lincoln, NE). The blot was then washed in triplicate with TBST and incubated for 1 hour with secondary antibody (1:10,000 dilution of IRDye 800 anti-mouse IgG and IRDye 680 anti-rabbit IgG, Li-cor Biosciences, Lincoln, NE) in antibody dilution buffer. After a final triplicate wash with TBST, the blot was visualized using the Odyssey CLx imaging system (Li-cor Biosciences, Lincoln, NE).

### Reverse transcription polymerase chain reaction (RT-PCR)

Samples were lysed in RLT buffer (Qiagen, Hilden, Germany) + 0.1% β-mercaptoethanol, transferred to individual QIAshredders (Qiagen, Hilden, Germany) and homogenized via centrifugation at 17000 x g for 15 minutes. The supernatant was collected, and total RNA was extracted using the RNeasy mini kit (Qiagen, Hilden, Germany) and RNase-free DNase set (Qiagen, Hilden, Germany) following the manufacturers protocol. Total RNA concentration was determined using the Nanodrop 8000 spectrophotometer (Thermo Fisher Scientific, Waltham, MA) and reverse transcription was performed using the High Capacity cDNA Reverse Transcription kit (Applied Biosystems, Waltham, MA) using up to 2μg of RNA as a template. 50ng of the resulting cDNA product was subjected to duplex PCR reactions using Gene Expression Master Mix (Applied Biosystems, Waltham, MA) containing Taqman primers for TTBK1 (Cat# Mm01269698), TTBK2 (Cat# Mm00453709), and housekeeping gene GAPDH (Cat# Mm99999915, all primers are from Applied Biosystems, Waltham, MA). Real-time PCR reactions were performed on the Via7 Real-time PCR system (Thermo Fisher Scientific, Waltham, MA) using the following thermocycling conditions: 2 min at 50°C, 10 min at 95°C, followed by 40 cycles of 15 sec at 95°C and 1 min at 60°C. Relative gene expression levels of TTBK1 and TTBK2 were calculated using the ΔΔCt method.

### Microtubule sedimentation

All steps of the microtubule sedimentation assay were performed at 37°C to maintain the microtubule network of the samples. Samples were lysed at 37°C for 10 min in warm 1:1 Lysis and Microtubule Stabilization Buffer (Cytoskeleton Inc., Denver, CO) and water, supplemented with 20μM taxol, 1mM ATP, 100μM GTP, and 1% protease inhibitor cocktail (Cytoskeleton Inc., Denver, CO). The lysate was syringe sheared ten times and spun at 2000 x g at 37°C for 5 min to clear out debris. The pre-cleared supernatant was then subjected to ultracentrifugation at 100,000 x g for 1 hour at 37°C to separate the soluble fraction and the microtubule pellet. These fractions were denatured and analyzed for relevant proteins using SDS-PAGE.

### Microtubule polymerization

Co-transfected HEK293 cells or recombinant proteins were lysed in cold General Tubulin Buffer (Cytoskeleton Inc., Denver, CO) with 0.1% Triton X100, 0.1% Tween20, and 1% Halt protease and phosphatase inhibitor cocktail (Thermo Fisher Scientific, Waltham, MA) for 30 min at 4°C before being centrifuged at 17,000 x g for 15 min at 4°C to pellet debris. Protein concentrations were determined using a Direct Detect Infrared Spectrometer (EMD Millipore Corp., Burlington, MA) and 10μl of protein at 1.5μg/μl were loaded into each well of a 96-well black coated assay plate (Corning Inc., Corning, NY). 50μl of tubulin polymerization mix, consisting of 205μl fluorescent reporter Buffer 1, 150μl Tubulin Glycerol buffer, 4.4μl 100mM GTP, and 85μl 10 mg/ml tubulin (all reagents were adapted from the Tubulin Polymerization Assay kit, Cat# BK011P, Cytoskeleton Inc., Denver, CO). The plate was transferred to a Spectramax M2 microplate reader (Molecular Devices, San Jose, CA) set at 37°C and the rate of microtubule polymerization was determined by measuring fluorescence (excitation at 360 nm, emission at 450 nm) every minute for 60 minutes.

### Cell assay investigating tau phosphorylation at S422

HEK293 cells were transfected with plasmids encoding Tau and TTBK1 were seeded 24 hours later into 384-well plates at 10,000 cells per well. After an additional 24 hours, the transfected cells were treated with compounds or DMSO and incubated at 37°C for an hour. Cell were lysed in RIPA buffer supplemented with protease and phosphatase inhibitors (Thermo Fisher Scientific, Waltham, MA). Phosphorylation of Tau at residue S422 was measured by FRET assay using the anti-Tau and anti-phospho S442 Tau antibodies conjugated with d2 (acceptor) and Tb cryptate (donor), respectively (Cisbio Bioassays, Bedford, MA). The fluorescence level was measured using an EnVision plate reader (Perkin Elmer, Waltham, MA).

### Animal use

All experiments were conducted in compliance with the rules set forth by the Biogen Institutional Animal Use and Care Committee in accordance with the guidelines established in the National Institutes of Health *Guide for the Care and Use of Laboratory Animals*. For *in vivo* experiments, subjects were anesthetized with isoflurane to induce hypothermia and were sacrificed after an hour. Subjects were kept under anesthesia the entire duration of hypothermia to ensure no pain or distress. These protocols were approved by the Biogen IACUC committee.

### Primary mouse neuron culture

Primary cultures of mouse neurons were prepared using the brains of E18, CD-1 mouse embryos. Following decapitation, the embryo brains were extracted and transferred to a dish with ice-cold 20% FBS (Gibco, Waltham, MA) and HBS, consisting of Hank’s Balanced Salts (Sigma-Aldrich, St. Louis, MO), 0.35g/L NaHCO_3_, and 1mM Hepes (Gibco, Waltham, MA). The meninges were removed, and the cerebral cortices were dissected from the rest of the brain. The cortices were finely chopped, washed in triplicate with ice-cold HBS, and enzymatically digested with 5 mls of digestion buffer (8g/L NaCl, 0.27g/L KCl, 0.99mg/L Na_2_HPO_4_, 25mM Hepes), plus 4mg of trypsin (Sigma-Aldrich, St. Louis, MO) per cortex and 4.5KU Dnase I (Sigma-Aldrich, St. Louis, MO) for 10 min at 37°C. The digested neurons were washed three times with HBS + 20% FBS, followed by three washes with HBS. The cells were dissociated in 5mls of trituration buffer (HBS with 2.95g/L MgSO_4_7H20) plus 4.5KU DNase I (Sigma-Aldrich, St. Louis, MO) using 60 strokes of a 1ml pipette tip and spun at 300 x g for 10 min at 4C. The trituration buffer was aspirated, and the pellet was resuspended in culture media, consisting of Minimum Essential Media (Gibco, Waltham, MA) with 5g/L glucose, 0.2g/L NaHCO_3_, 0.1g/L transferrin (EMD Millipore Corp., Burlington, MA), 5% FBS, 500μM L-glutamine (Gibco, Waltham, MA), 2% B-27 (Gibco, Waltham, MA), 10 units/ml penicillin and 10μg/ml streptomycin (Gibco, Waltham, MA). After filtering out cell debris using a 40μM filter (Corning Inc., Corning, NY), 175,000 live cells were plated into each well of a 12-well pre-coated poly-D-lysine plate (Corning Inc., Corning, NY) and incubated at 37°C in a humidified 5% (v/v) CO_2_/air environment. Every subsequent two days, the media was supplemented with an additional quarter volume of fresh culture media for maintenance. For lentiviral knockdown experiments, two TTBK1 shRNA sequences were cloned into the pLKO1 backbone (TTBK1 shRNA sequence 1: CTACTTCACCAAGCCCGATTA; TTBK1 shRNA sequence 2: ACATCAAGCCGTCCAACTTTG). Lentivirus was made in HEK293T cells and added onto primary neurons at DIV2.

### Hypothermia induced tau phosphorylation

Seven-week-old male C576Bl/6 mice were used for all hypothermia experiments (Charles River Laboratories, Wilmington, MA). Hypothermia was induced by exposure to vaporized isoflurane in an anesthetic induction chamber attached to a V-1 Tabletop Anesthesia System (VetEquip, Inc., Livermore, CA). The mice remained in the chamber for 60 minutes with a constant isoflurane concentration of 2%. Temperature measurements were obtained via rectal probe (Thermalert TH-5; Physitemp, Clifton, NJ) immediately before and after anesthesia to ensure successful induction of hypothermia. Normally the animals had a body temperature drop to a mean of 27.6°C over the one hour of anesthesia.

### Blood and tissue collection and preparation

Following hypothermia induction, 200–300 μL of blood was collected via cardiac punch and transferred to K_2_EDTA-treated BD Microtainers (Becton Dickinson and Co., Franklin Lakes, NJ). Mice were sacrificed via decapitation and brains were quickly removed and bisected sagitally. The right cortices, were isolated, placed into 2 mL microtubes along with a single 5 mm stainless steel bead and the left cortices were placed into 2 mL Lysing Matrix A tubes (MP Biomedicals, Santa Ana, CA). Both tubes were immediately snap frozen in liquid nitrogen. The whole blood-containing microtainers were centrifuged (2000 x g, 5 minutes) and plasma was transferred to 96-well plates for pharmacokinetic determination. Microtubes containing the right cortices and beads were placed into a TissueLyser II (Qiagen, Hilden, Germany) with Pierce RIPA buffer (Thermo Fisher Scientific, Waltham, MA) + Halt Protease and Phosphatase Inhibitor Cocktail (Thermo Scientific, Waltham, MA) + PhosSTOP (Roche, Basel, Switzerland) + cOmplete Mini (Roche, Basel, Switzerland) + PMSF (Cell Signaling Technology, Inc., Danvers, MA) in a 5:1 ratio of buffer volume to tissue weight. The samples were shaken at 30 Hz for 2 minutes. Upon cycle completion, lysates were transferred to individual QIAshredders (Qiagen, Hilden, Germany) and centrifuged at 17000 x g for 15 minutes. Following centrifugation, the supernatant was carefully transferred to a set of fresh 1.5 mL microcentrifuge tubes. The protein content of each sample was quantified using a Direct Detect Infrared Spectrometer (EMD Millipore Corp., Burlington, MA).

### Statistical methods

Linear regression was performed to investigate that relationships between free drug concentration (nM) and either target engagement (measured by the percentage of ATP/ADP peptide probe inhibition) or the phosphorylation level at S422 (represented as a percentage of the vehicle treated control). In the hypothermia model the level of tau phosphorylation at each epitope was normalized to the amount of total tau within a sample. An ordinary one-way ANOVA was used to test for a drug treatment effect on the level of tau phosphorylation at each epitope separately. A Tukey’s multiple comparisons test was used to look for effects between groups–using the vehicle treated hypothermia group as the control.

## Supporting information

S1 Methods (DOCX)

S1 Table Staurosporine IC50 for common tau kinases. (TIF)

S1 Fig Western blot demonstrating tau phosphorylation prior to tubulin polymerization assay. (TIF)

S2 Fig IC50 of BIIB-TTBK1i in biochemical assay. (TIF)

S3 Fig In vivo phamacokinetic profile of BIIB-TTBK1i. (TIF)

S4 Fig Significant increase in TTBK1 transcript in AD brains. (TIF)

S5 Fig Targeted proteomics approach demonstrates no change in TTBK1 protein expression in AD brains post-mortem. (TIF)

S1 Raw Images (PDF)
