# Supplementary material for: Acute inhibition of the CNS-specific kinase TTBK1 significantly lowers tau phosphorylation at several disease relevant sites
Source: PLoS One. 2020 Apr 7;15(4):e0228771. doi: 10.1371/journal.pone.0228771 (PMC7138307; doi:10.1371/journal.pone.0228771)
Supplement: S1 Methods — (DOCX) [file pone.0228771.s001.docx]

# Supplemental Methods:

## TTBK biochemical Assay:

## Kinase reactions were performed in a total volume of 30 µL in 384-well plates (TTBK1 1-479aa; SignalChem Catalog # T17-11G-05). Final Kinase Activity conditions; 400 pM TTBK1 at ambient temperature in 25 mM Hepes, pH 7.5, 50 mM NaCl, 10 mM MgCl2, 0.02% Brij-35, 1 mM DTT, 10 uM ATP and 100 nM Ulight TOPO IIa peptide substrate (FDEKTDDEDF). After a 2 hour incubation at 23 °C, the kinase reaction was quenched with 30 ul of 60 mM EDTA, and 2 nM Eu-anti- FDEKpTDDEDF antibody in 1X Lance Delfia Detection Buffer and incubated for one hour prior to detection with the EnVision Multilabel Reader (PerkinElmer). Fluorescence signals were read using an excitation filter of 320 nM and emission filters of 665 nm and 615 nm. Activity was expressed as the Acceptor/Donor (665 nm/615 nm) signal ratio. For compound inhibition, 15 ul of 800 pM TTBK1 was preincubated for 30 minutes with 300 nl of 100x compound stock in DMSO before initiation of the activity by addition of 15 ul of 2x Substrate Stock (200 nM peptide and 20 uM ATP).

## Drug Metabolism and Pharmacokinetics

*In vitro* plasma and brain protein binding values were generated via the Rapid Equilibrium Dialysis method. The compound of interest was incubated in K2EDTA mouse plasma and rat brain homogenate (homogenized 1:7 (w:v) in 1xPBS) purchased from BioIVT, opposite a buffered compartment of 100mM Potassium phosphate/150mM Sodium chloride, pH 7.4, at 1uM for 4hr and 6hr respectively. At the conclusion of incubation, samples were taken from both matrix and buffered compartments, matrix-matched using blank buffer and matrix, extracted with acetonitrile, diluted with water, and analyzed utilizing an Agilent RapidFire 365 high-throughput LC coupled with MS/MS detection via an AB Sciex 5500. Free fractions (fu) were then calculated by comparing peak-area ratios of matrix and buffered compartments. Cross-species brain protein binding was considered to be equivalent for the purposes of calculating free fraction (Di L et al., 2011).

*In vivo* pharmacokinetic studies were conducted in accordance with Biogen IACUC guidelines in 20-25g C57BL/6 mice purchased from Charles River Labs. A total of 9 mice, split into 3 groups (A,B,C), were dosed subcutaneously at 30mg/kg (3mg/mL; 10mL/kg) in a vehicle of 20% Captisol. Approximately 50uL of blood was collected from the mice at each timepoint by submandibular bleed into K2EDTA tubes according to a staggered study design – 15min(A), 30min(B), 1hr(C), 3hr(B), 5hr(C), 7hr(B), 16hr(A), 24hr(C). Terminal timepoint samples for each group (7hr, 16hr, 24hr) were taken post CO2 euthanasia via cardiac puncture. Blood samples were spun at 10,000rpm for 5 minutes at 4°C to generate plasma for total drug concentration measurement. Mouse brains were harvested for total drug concentration measurement, placed into MP Biomedicals Lysing Matrix D tubes, and stored at -80°C, along with plasma samples to await quantitative analysis.

Total drug concentration in plasma and brain tissue was measured via well-established bioanalytical extraction (protein precipitation) and detection methods (LC-MS/MS). Brain tissues were homogenized 1:4 (w:v) with 1x PBS in MP Biomedicals Lysing Matrix D tubes via an MP Biomedicals FastPrep-24™ homogenizer and were then extracted alongside plasma samples by matrix-matching with blank mouse K2EDTA plasma (purchased from BioIVT), followed by protein crash/extraction with acetonitrile, supernatant dry down under nitrogen, and reconstitution with an acidified aqueous/organic mixture before being measured against a calibration curve of the compound of interest prepared in plasma, matrix-matched with blank mouse brain homogenate (generated with brains purchased from BioIVT), and similarly extracted. Reconstituted extracts were then analyzed via LC-MS/MS (AB Sciex 5500) utilizing a binary HPLC setup (Shimadzu LC-20ADvp) and reverse-phase chromatography gradient (ACE 3 C18-AR). Peak area ratios and a 1/x2 regression fit were used to generate sample concentration values that, combined with plasma and brain protein binding values, were used to generate free drug concentration values and profile.

## Nanostring:

*Gene expression profiling*

Control and Alzheimer’s disease brain samples were supplied by the Netherlands brain bank (Superior Frontal Gyrus; Control n=11, AD n=10; Average Braak Stage 6). Total RNA was isolated using RNeasy kit (Qiagen) according to manufacturer’s protocol. 250ng RNA per sample was used for gene expression profiling using nCounter Analysis (NanoString technology). Selection of the Nanostring probeset was based on genes proposed to be relevant to AD pathophysiology and included 75 disease-associated genes and 5 housekeeping genes.

*Data normalization and analysis*

NanoString data were normalized and analyzed using nSolver™ software. RNA ncounts were normalized using the geometric mean of five housekeeping genes including GAPDH, G6PD, TBP, TUBB, and HPRT1 using nSolver™ Analysis Software, version 3.0 (NanoString Technologies, Inc.). A cutoff was introduced at the value two‐fold of the highest negative control present on the chip. Fold changes were calculated using the average of each group. For each experiment, the fold changes were calculated comparing the experimental group to their appropriate controls..

## Targeted Mass Spec:

*Tissue sample preparation*

Brain tissue was procured from University of Miami Brain Bank. Brain samples (Human Biological Samples) supplied by the University of Miami Brain Bank were obtained using informed consent form that approves the use of Human Biological Samples for research purposes. Hippocampal tissue from 10 patients with Braak III-IV stage of Alzheimer’s was used along with hippocampal tissue from 10 age-matched unaffected (healthy) individuals. Samples were lysed in Tris homogenization buffer (10mM Tris pH7.4, 0.8mM NaCl, 1mM EGTA, 1mM Na3VO4, 1mM NaF, 10% sucrose, supplemented with HALT protease and phosphatase inhibitors (ThermoFisher Scientific)), and homogenized using TissueLyzer (Qiagen). The samples were centrifuged and the pellet was reextracted with Tris homogenization buffer one more time and centrifuged again. The two supernatant were combined and solid urea was added to each sample for a final 8M concentration. Prior to tryptic digestion, protein concentration was determined by BCA protein assay (ThermoFisher Pierce, Rockford, IL). Stock solution of 500 mM DTT was added to the samples to make a final concentration of 5 mM followed by incubation at 37 °C for 1 h with shaking at a speed of 1,200 r.p.m. Following reduction, stock solution of 400 mM IAA was added to make a final concentration of 10 mM followed by incubation at room temperature for 1 h in the dark with shaking at a speed of 1,200 r.p.m. Samples were diluted 4x prior to trypsin addition at a ratio of 1:50 w/w of enzyme: protein followed by incubation at 37 °C for 12 h with shaking at a speed of 700 r.p.m. After digestion, the samples were acidified with 10% TFA to a final 0.5% TFA concentration. The samples were desalted using SPE cartridges (Waters). The SPE cartridge was prewashed with 2 ml of methanol and then with 2 ml of 0.1% TFA. The digest samples were slowly added through the cartridge. The cartridges were then washed with 4 ml of 5% acetonitrile with 0.1% TFA. Finally, the digest peptides were eluted with 1 ml of 80% ACN/0.1% TFA and completely lyophilized in speed vac.

*LC-SRM for protein quantitation*

The target peptide sequences of TTBK1, Actin and GAPDH proteins were selected based on public data repositories, and our own unpublished data. LC-SRM analysis was performed on a Easy nanoLC 1200 system (Thermo Scientific, San Jose, CA) interfaced to QExactive HF mass spectrometer (Thermo Scientific, San Jose, CA) equipped with an Easy nano-ESI source. An injection volume of 6 μl corresponding to 1ug of peptide digest was used for the analysis. Separation was performed using an Easy nano column PepMap II (75 μm id×500 mm, 1.7 μm, Thermo). The mobile phase A was 0.1% formic acid in water and mobile phase B was 0.1% formic acid in 80% acetonitrile. To achieve separation the following flow gradient at 250nL/min was used: starting at 2% solvent B ramping of 2–5% solvent B for 0–5 min, ramping of 5–30% solvent B for 5–100 min, ramping of 30–45% solvent B for 100-116min, ramping of 45–98% solvent B for 116-121 min, column washing for 5 minutes of 98–98% solvent B, and equilibrating for 8 minutes at 2% solvent A from 132-140 min. The QExactive HF mass spectrometer was operated in positive ion-mode with the ESI voltage set to 2,800 V and a capillary temperature at 325 °C. The SRM experiment was programmed to conduct scheduled SRM assays in PRM mode including an inclusion list. The optimum collision energy was set for each peptide. The summary of SRM parameters for all the peptides is described in supplementary Table X, noting precursor ion masses, peptide charge, collision energy values and retention time windows for each peptide. The resolution was set at 30000 with AGC target of 5e5 ions and maximum IT time of 200ms. Quadruple isolation window was 1.0 m/z. Data was analyzed on Skyline software where resulting chromatographic peaks from each run were integrated and the integrated peak areas were used to determine relative amounts of each peptide per sample. Resulting amounts for TTBK1 peptides were normalized to Actin and GAPDH corresponding peptides and unpaired student t-test was ran for each TTBK1 peptide.
