# Supplementary figures and images for: Acute inhibition of the CNS-specific kinase TTBK1 significantly lowers tau phosphorylation at several disease relevant sites
Source: PLoS One. 2020 Apr 7;15(4):e0228771. doi: 10.1371/journal.pone.0228771 (PMC7138307; doi:10.1371/journal.pone.0228771)

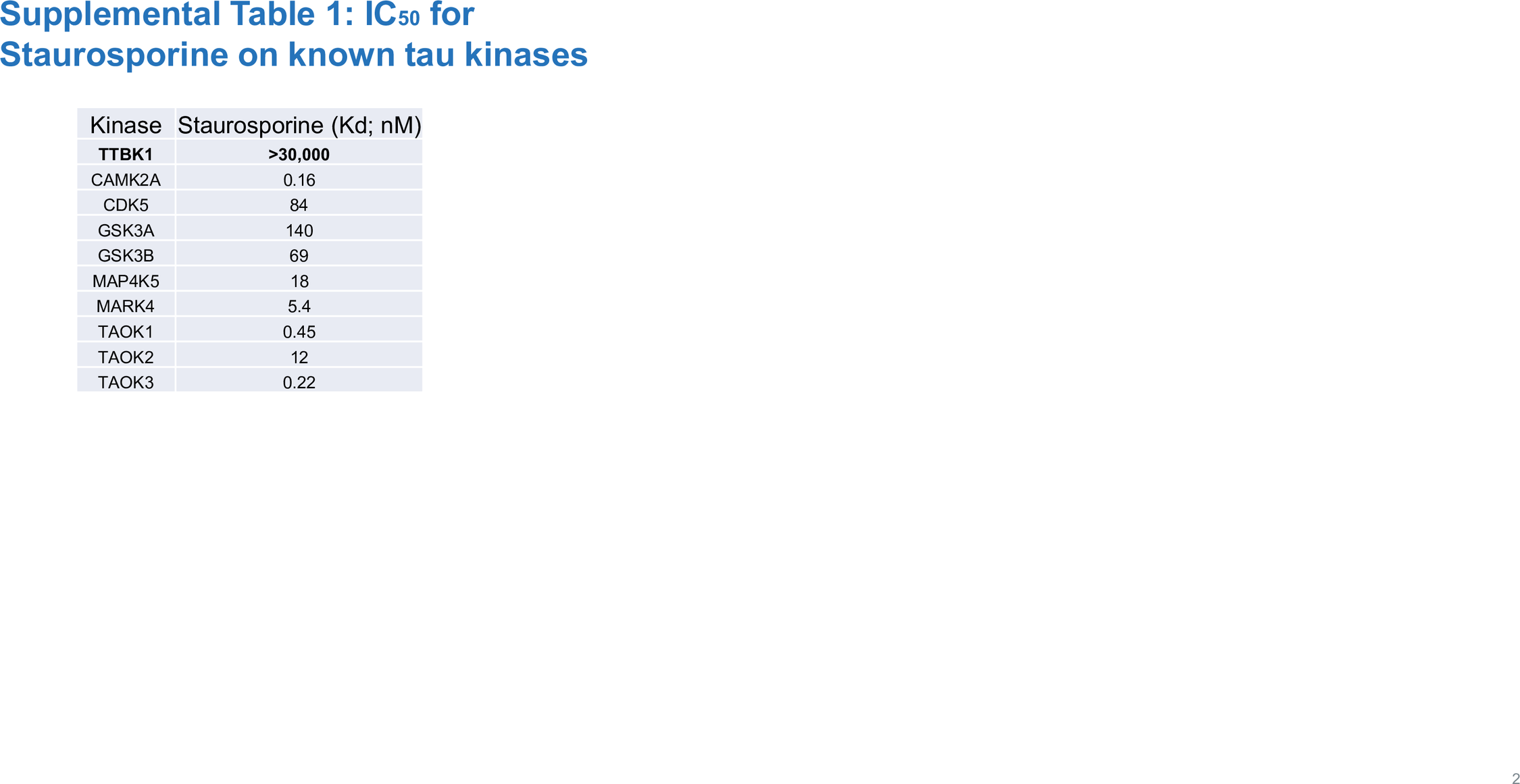

Supplement: S1 Table — (TIF) [file pone.0228771.s002.tif]

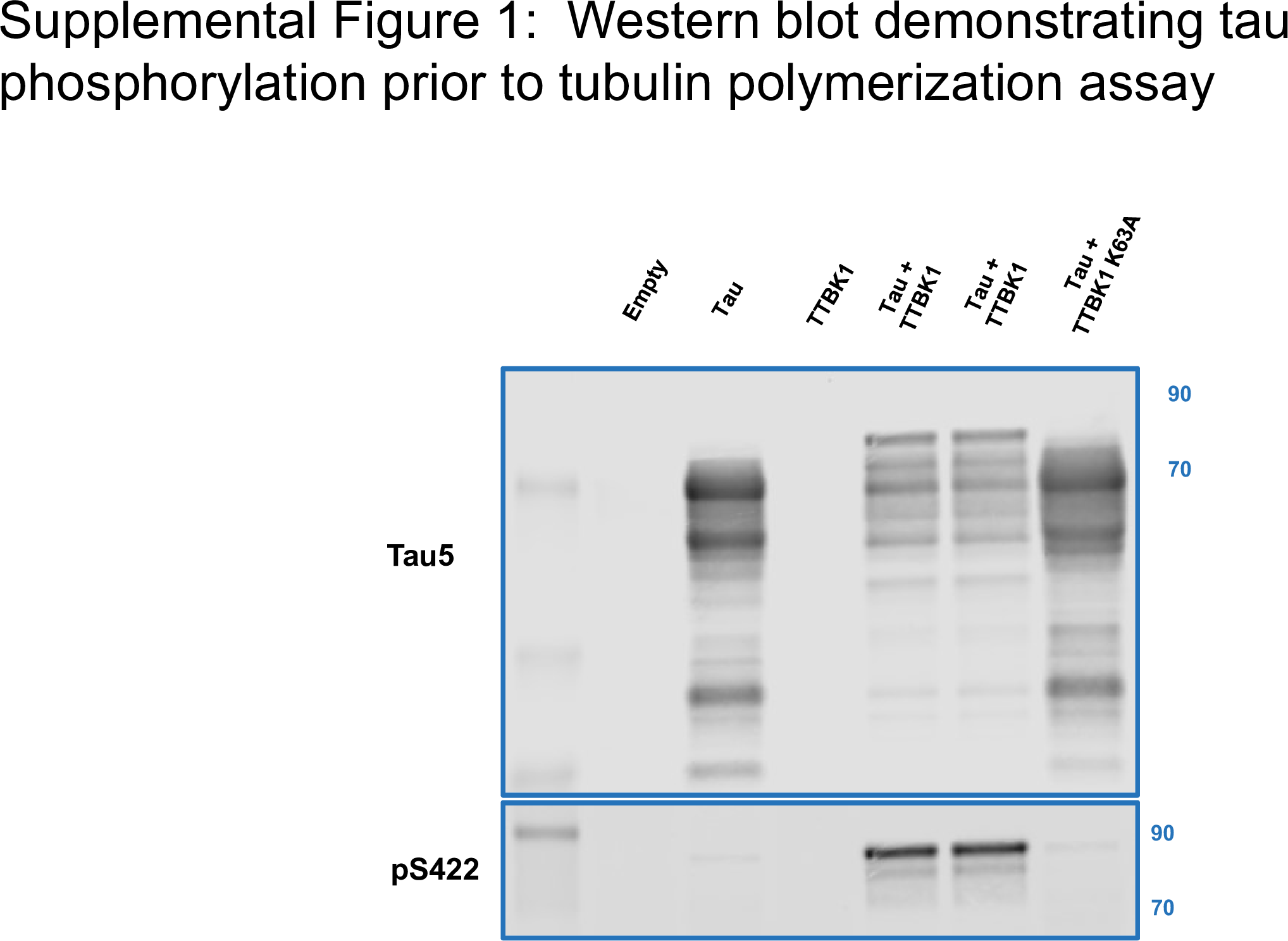

Supplement: S1 Fig — (TIF) [file pone.0228771.s003.tif]

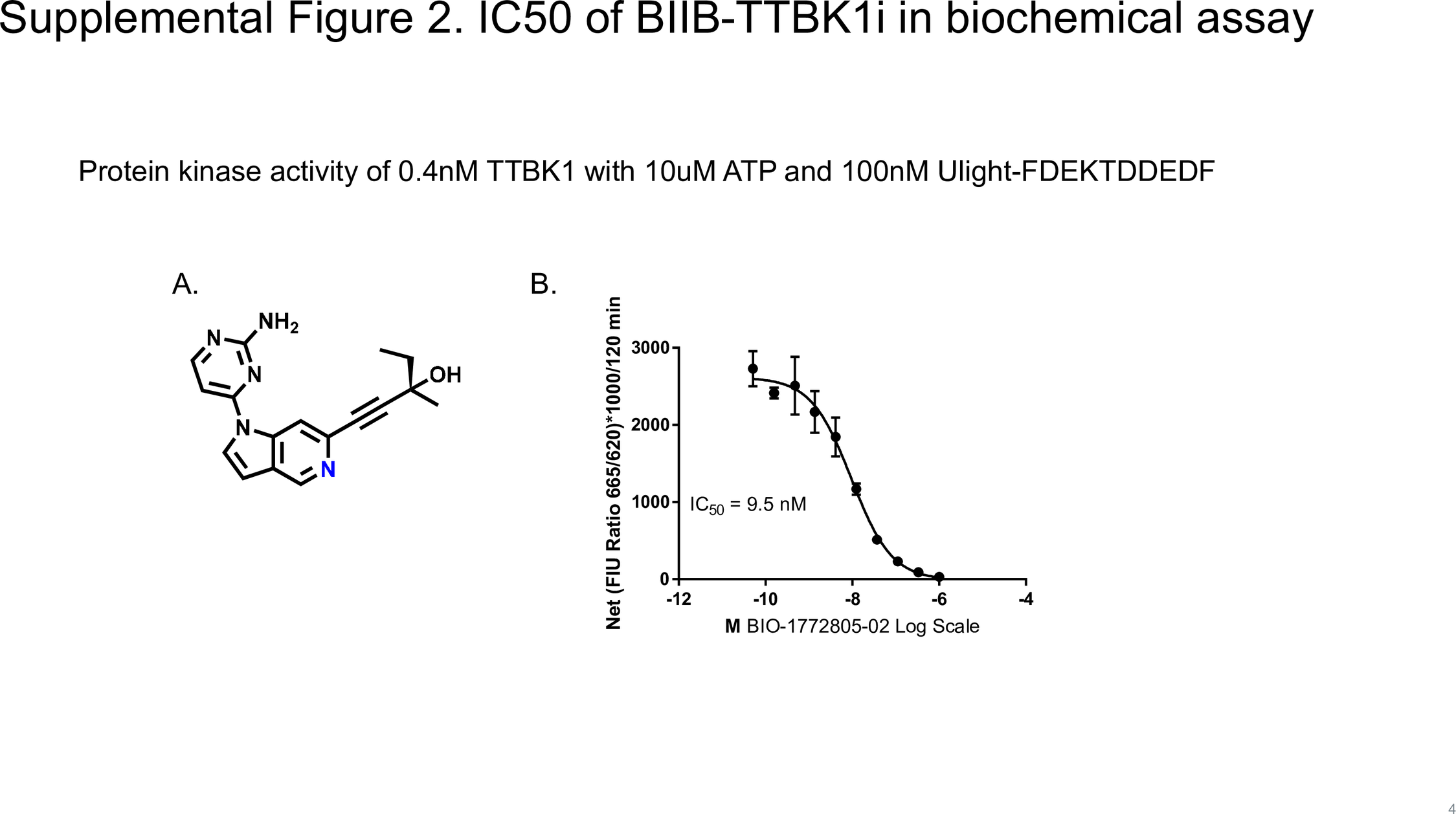

Supplement: S2 Fig — (TIF) [file pone.0228771.s004.tif]

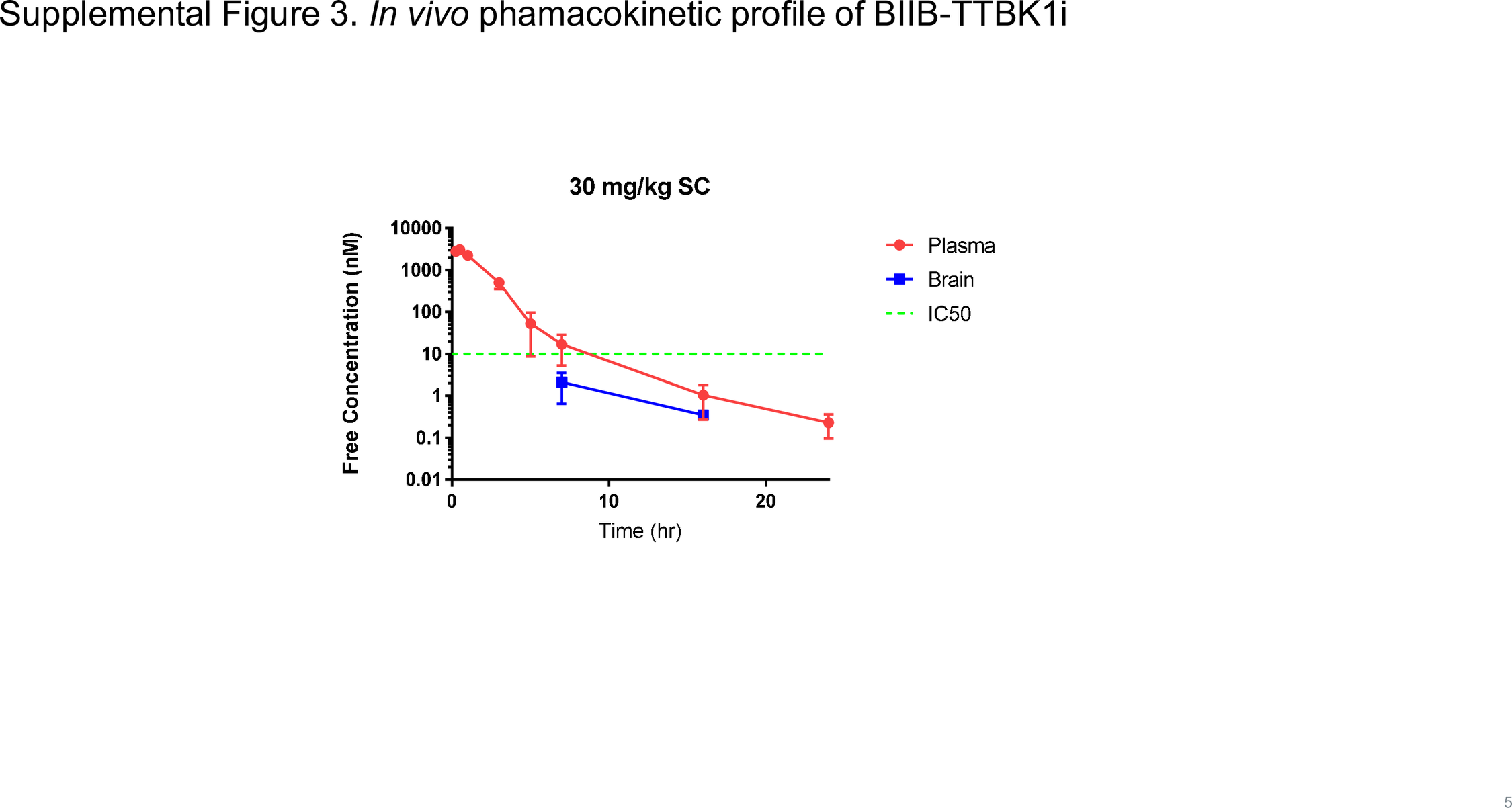

Supplement: S3 Fig — (TIF) [file pone.0228771.s005.tif]

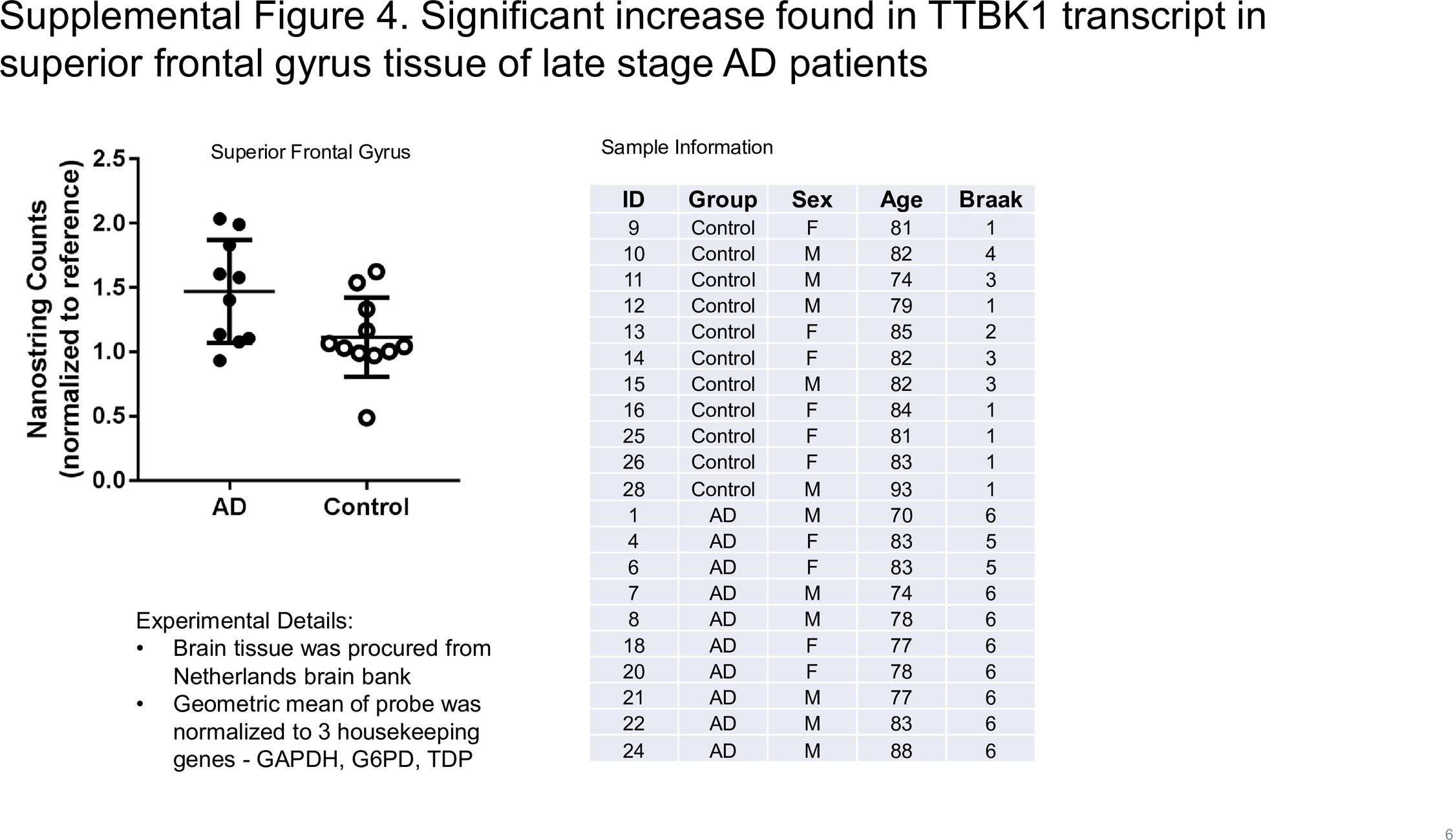

Supplement: S4 Fig — (TIF) [file pone.0228771.s006.tif]

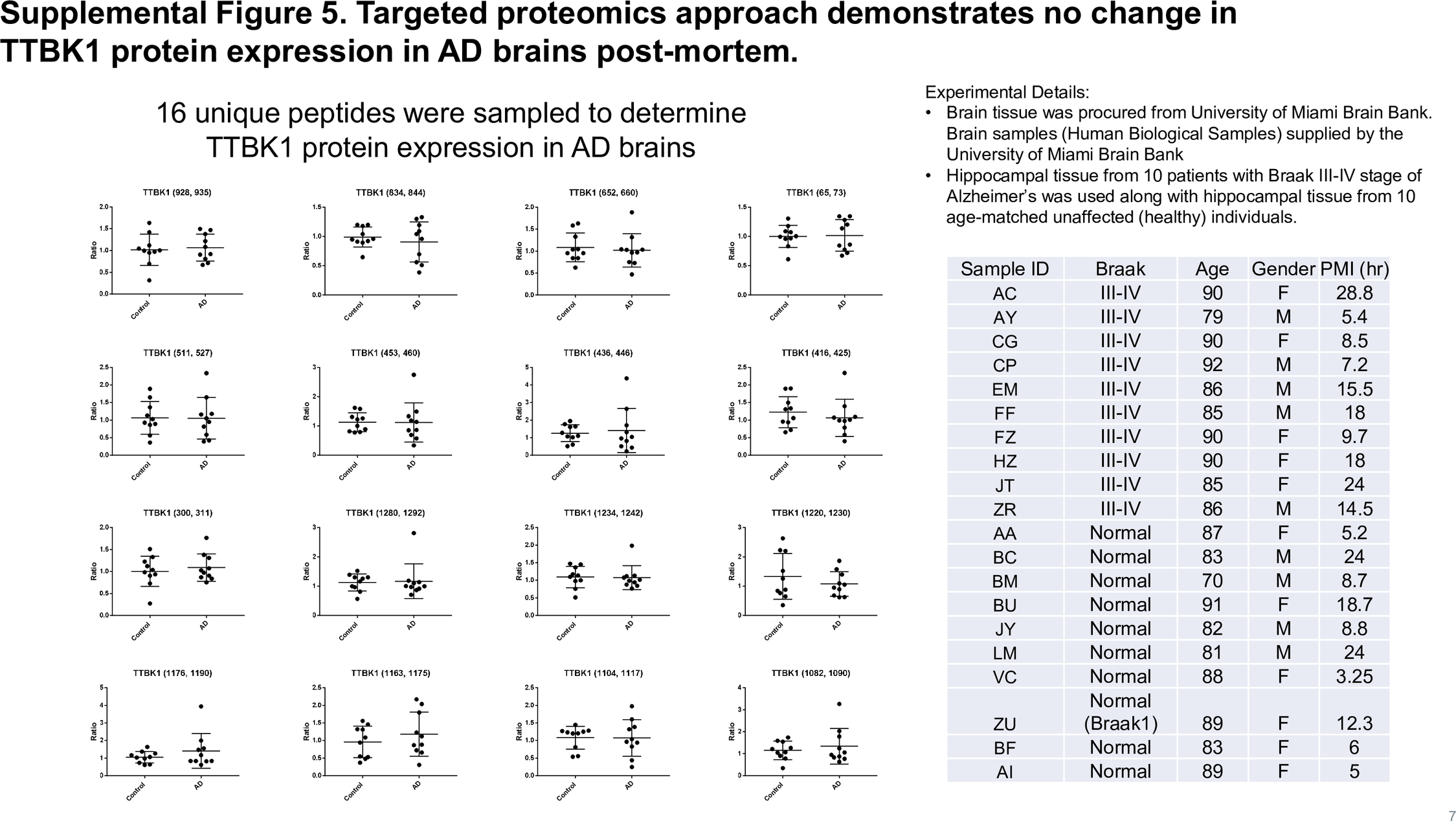

Supplement: S5 Fig — (TIF) [file pone.0228771.s007.tif]
