# Supplementary material for: Acute inhibition of the CNS-specific kinase TTBK1 significantly lowers tau phosphorylation at several disease relevant sites
Source: PLoS One. 2020 Apr 7;15(4):e0228771. doi: 10.1371/journal.pone.0228771 (PMC7138307; doi:10.1371/journal.pone.0228771)
Supplement: S1 Raw Images — (PDF) [file pone.0228771.s008.pdf]

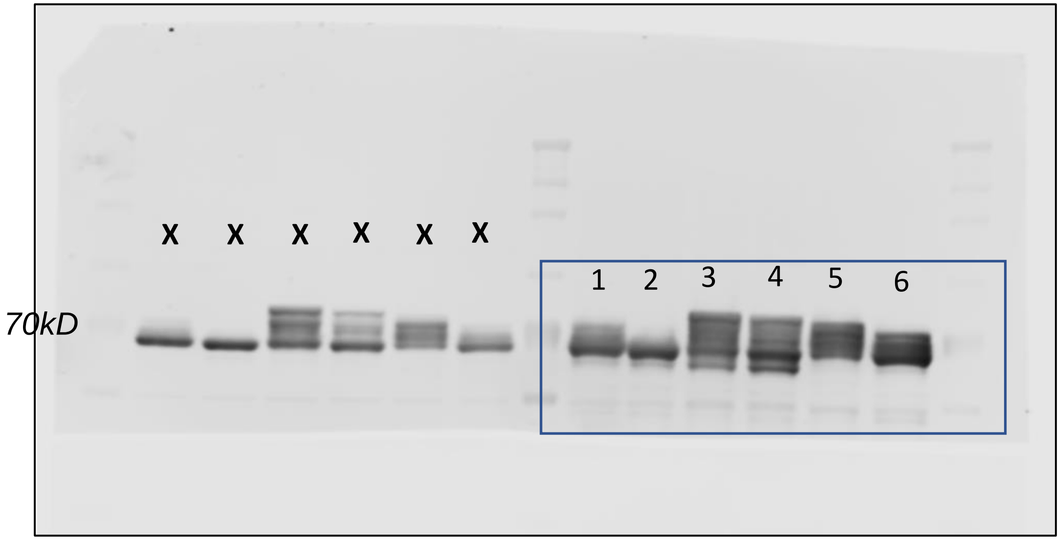


Figure 1B Tau 5

HEK cells transfected with following plasmids

1. Tau + Empty + Vehicle
2. Tau + Empty + Staurosporine 10uM
3. Tau + TTBK1 + Vehicle
4. Tau + TTBK1 + Staurosporine 10uM
5. Tau + GSK3B + Vehicle
6. Tau + GSK3B + Staurosporine 10uM

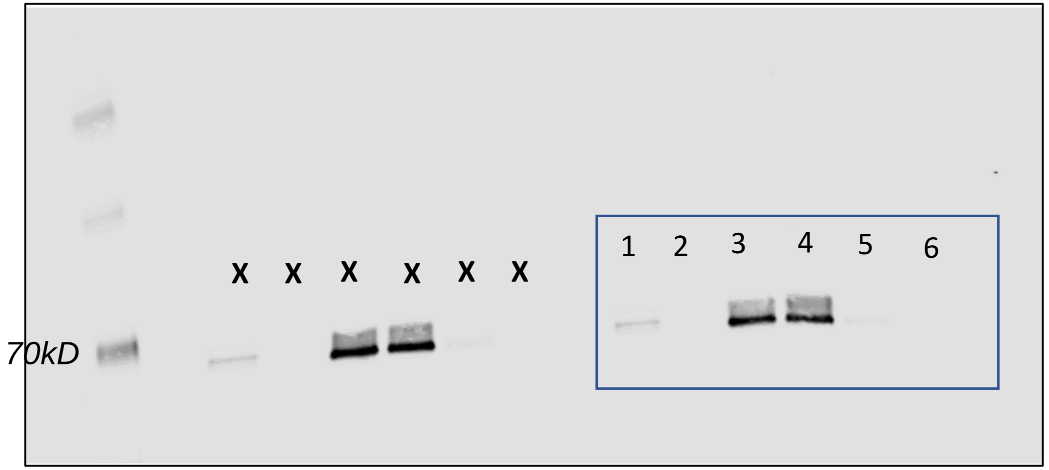


Figure 1B S422

HEK cells transfected with following plasmids

1. Tau + Empty + Vehicle
2. Tau + Empty + Staurosporine 10uM
3. Tau + TTBK1 + Vehicle
4. Tau + TTBK1 + Staurosporine 10uM
5. Tau + GSK3B + Vehicle
6. Tau + GSK3B + Staurosporine 10uM

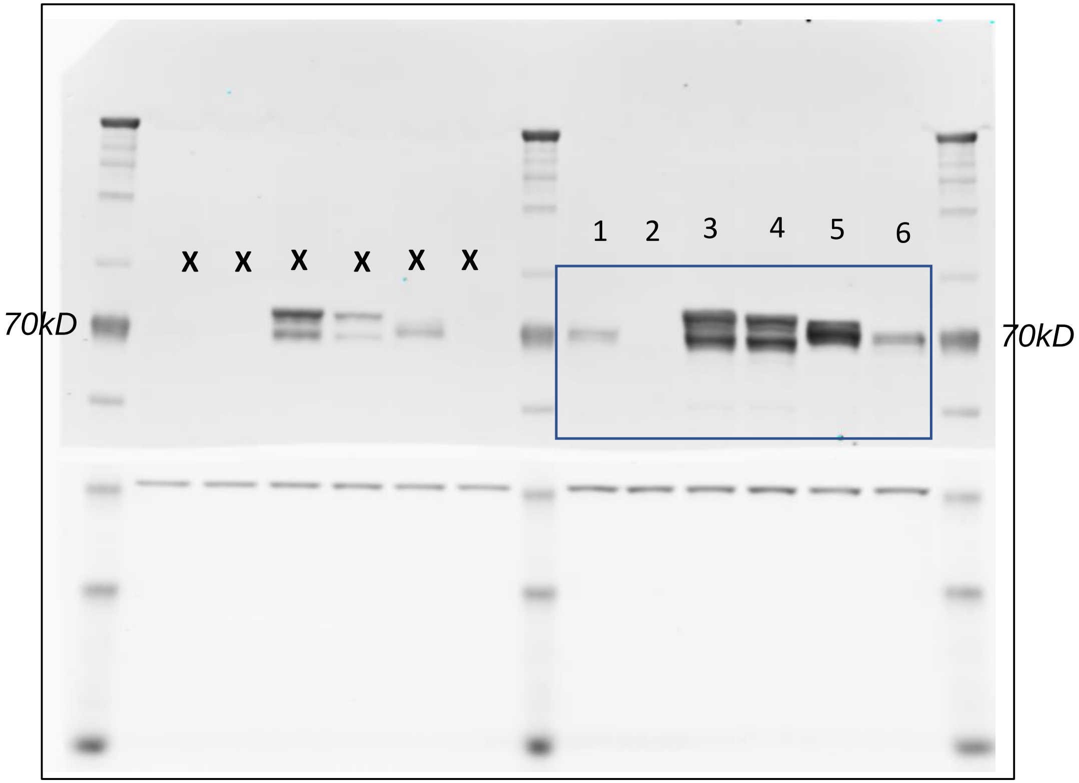


Figure 1B AT8

HEK cells transfected with following plasmids

1. Tau + Empty + Vehicle
2. Tau + Empty + Staurosporine 10uM
3. Tau + TTBK1 + Vehicle
4. Tau + TTBK1 + Staurosporine 10uM
5. Tau + GSK3B + Vehicle
6. Tau + GSK3B + Staurosporine 10uM

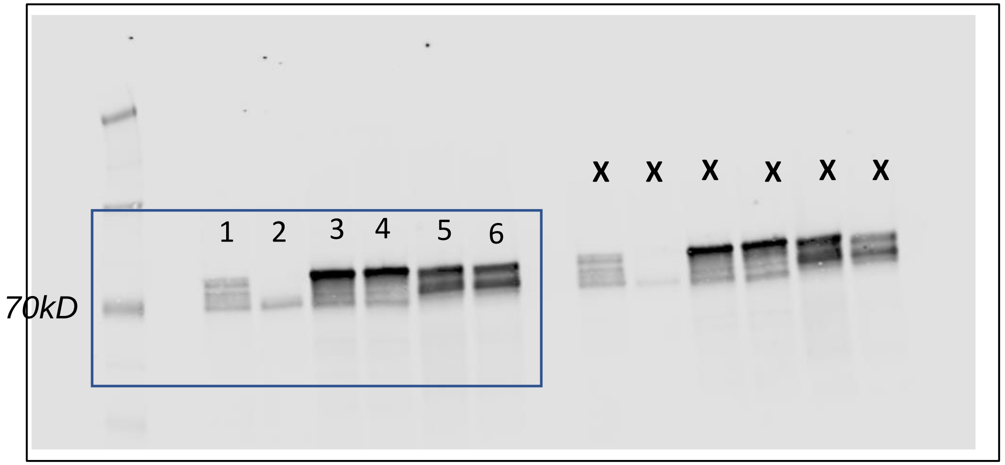


Figure 1B S198

HEK cells transfected with following plasmids

1. Tau + Empty + Vehicle
2. Tau + Empty + Staurosporine 10uM
3. Tau + TTBK1 + Vehicle
4. Tau + TTBK1 + Staurosporine 10uM
5. Tau + GSK3B + Vehicle
6. Tau + GSK3B + Staurosporine 10uM

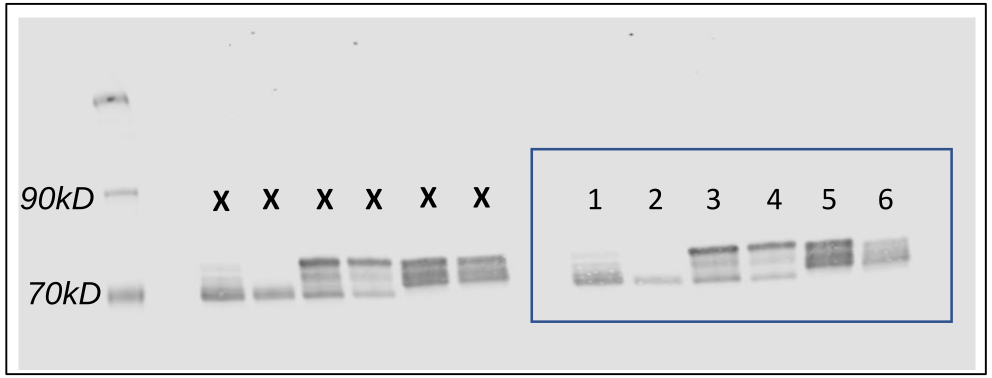


Figure 1B
Thr231
AT180

HEK cells transfected with following plasmids

1. Tau + Empty + Vehicle
2. Tau + Empty + Staurosporine 10uM
3. Tau + TTBK1 + Vehicle
4. Tau + TTBK1 + Staurosporine 10uM
5. Tau + GSK3B + Vehicle
6. Tau + GSK3B + Staurosporine 10uM

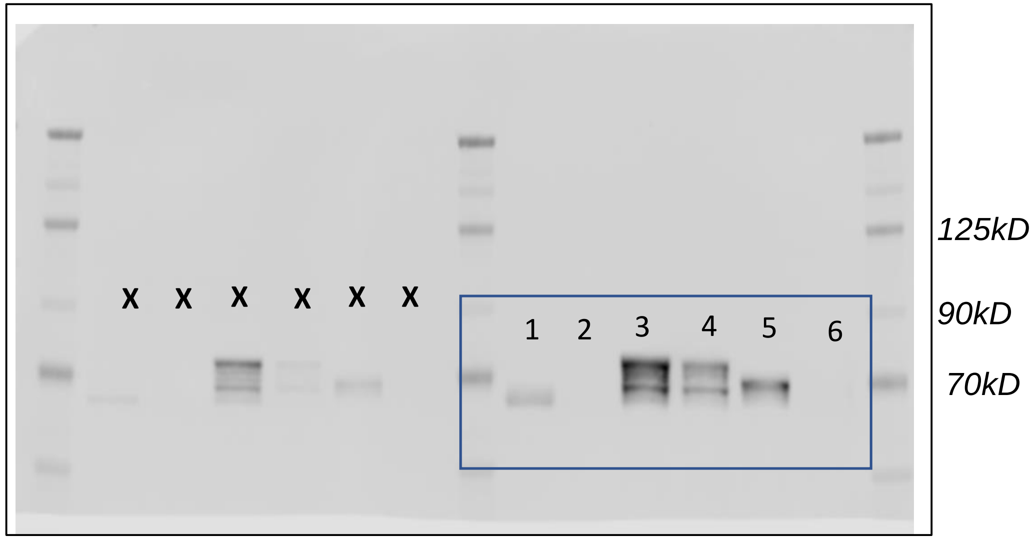


Figure 1B
pS214

HEK cells transfected with following plasmids

1. Tau + Empty + Vehicle
2. Tau + Empty + Staurosporine 10uM
3. Tau + TTBK1 + Vehicle
4. Tau + TTBK1 + Staurosporine 10uM
5. Tau + GSK3B + Vehicle
6. Tau + GSK3B + Staurosporine 10uM

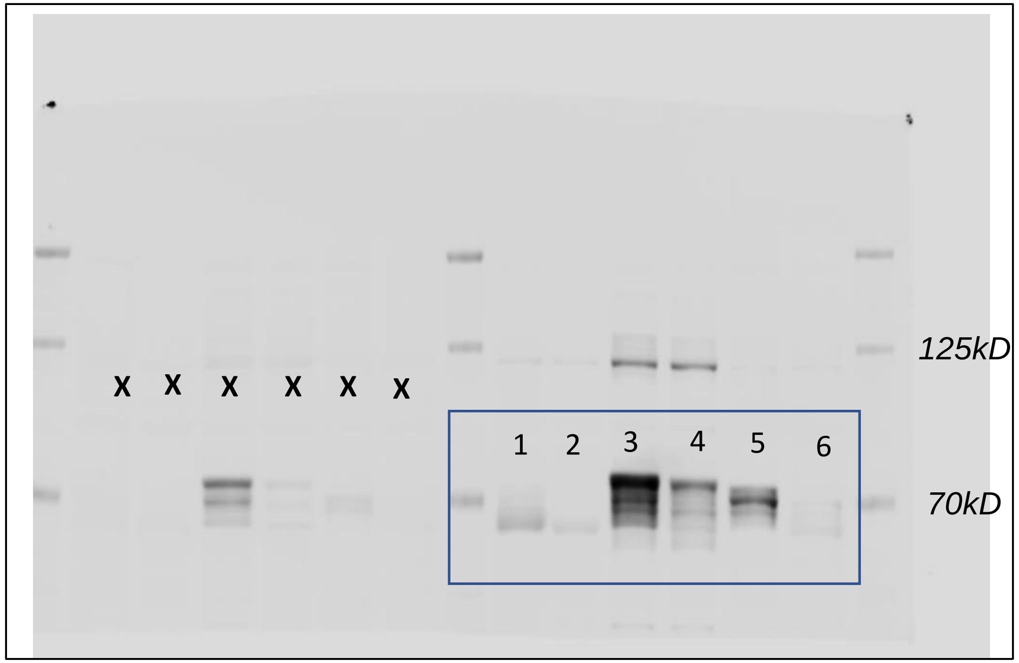


Figure 1B pS356

HEK cells transfected with following plasmids

1. Tau + Empty + Vehicle
2. Tau + Empty + Staurosporine 10uM
3. Tau + TTBK1 + Vehicle
4. Tau + TTBK1 + Staurosporine 10uM
5. Tau + GSK3B + Vehicle
6. Tau + GSK3B + Staurosporine 10uM

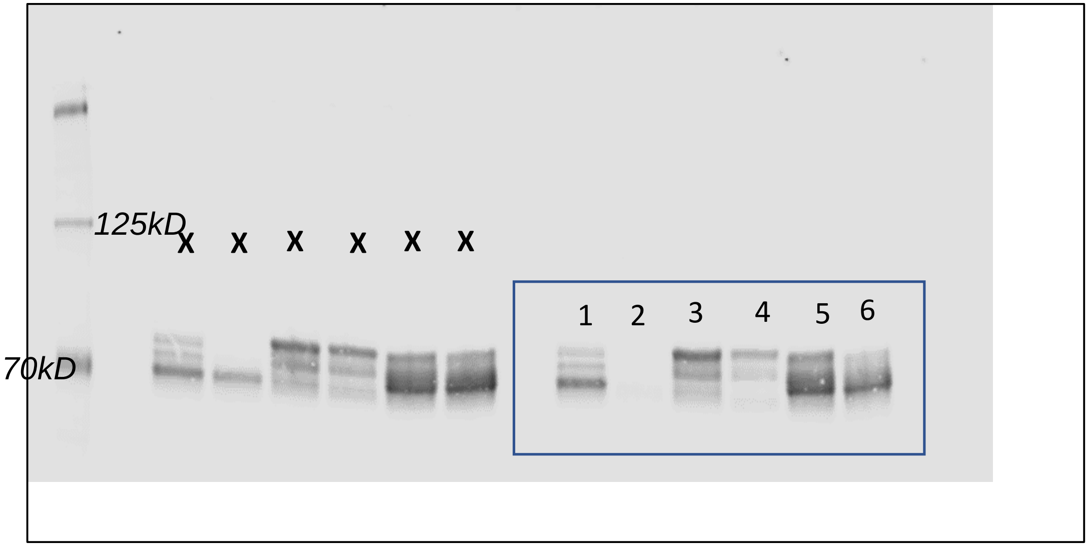


Figure 1B pS396 PHF-13

HEK cells transfected with following plasmids

1. Tau + Empty + Vehicle
2. Tau + Empty + Staurosporine 10uM
3. Tau + TTBK1 + Vehicle
4. Tau + TTBK1 + Staurosporine 10uM
5. Tau + GSK3B + Vehicle
6. Tau + GSK3B + Staurosporine 10uM

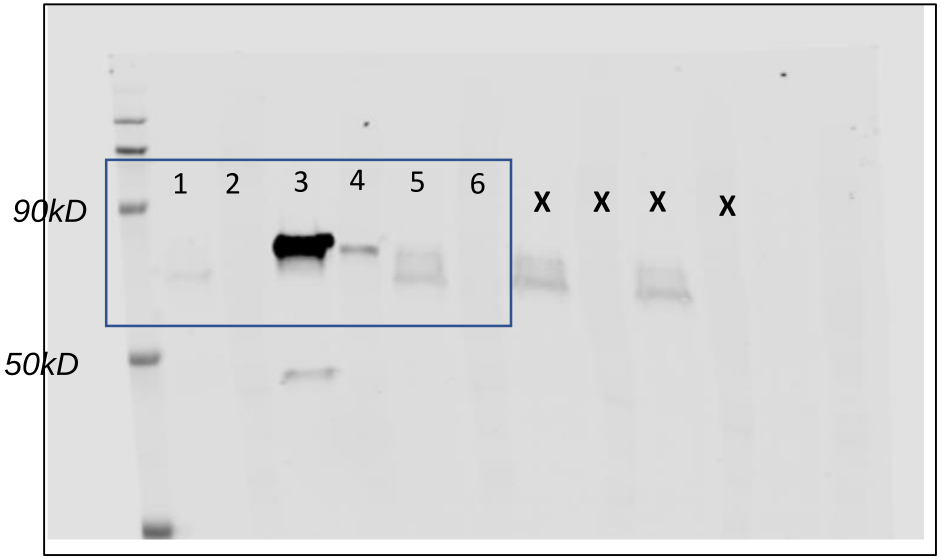


Figure 2A S422

HEK cells transfected with following plasmids and then resulting fractionation

1. Tau + Empty – Soluble fraction
2. Tau + Empty – Pellet Fraction
3. Tau + TTBK1 – Soluble fraction
4. Tau + TTBK1 – Pellet Fraction
5. Tau + TTBK1 (K63A) – Soluble fraction
6. Tau + TTBK1 (K63A) – Pellet Fraction

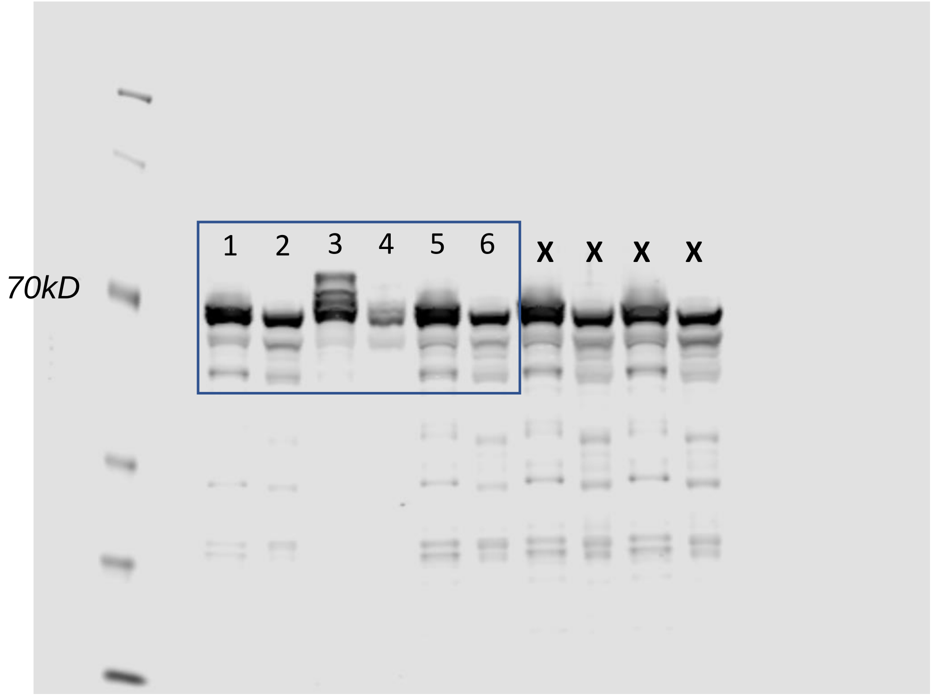


Figure 2A Tau5

# 3 separate experiments to get quantification of blots for Figure 2A

Experiment 1

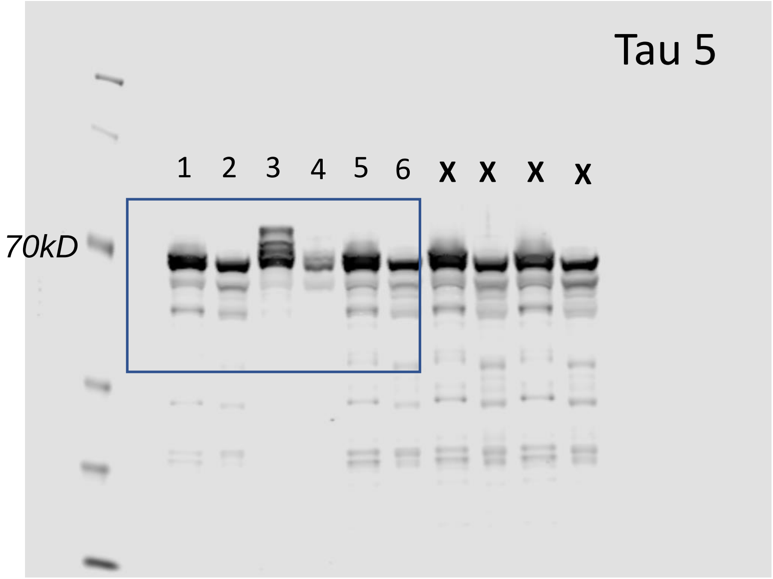


Experiment 2

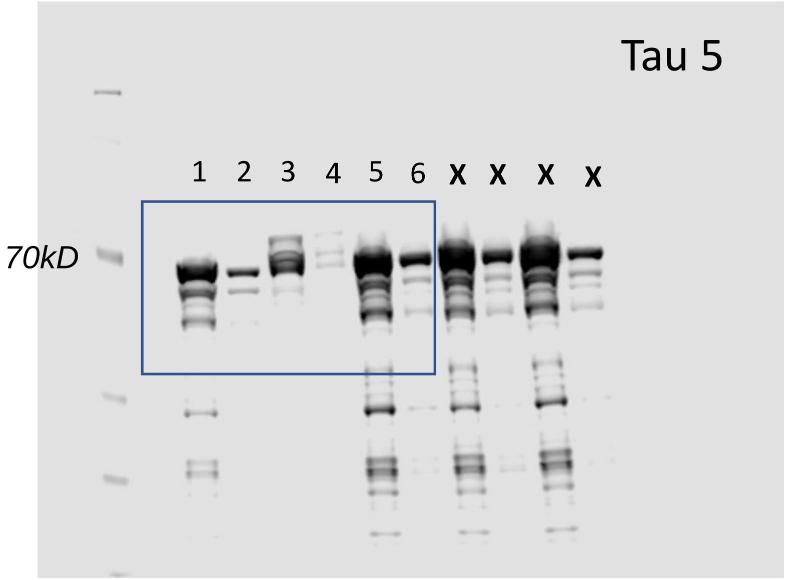


Experiment 3

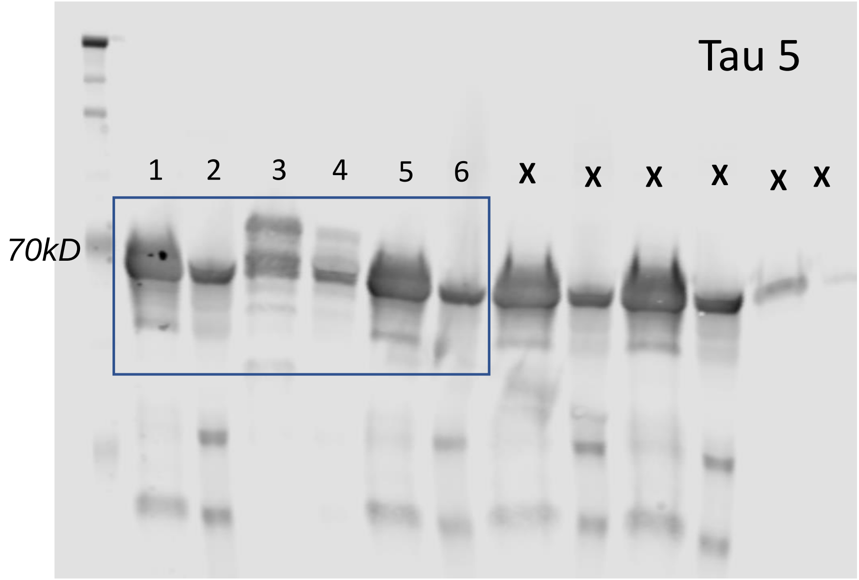


HEK cells transfected with following plasmids and then resulting fractionation

1. Tau + Empty – Soluble fraction
2. Tau + Empty – Pellet Fraction
3. Tau + TTBK1 – Soluble fraction
4. Tau + TTBK1 – Pellet Fraction
5. Tau + TTBK1 (K63A) – Soluble fraction
6. Tau + TTBK1 (K63A) – Pellet Fraction

Quantification done based on band intensity from Licor

Figure 3B S422

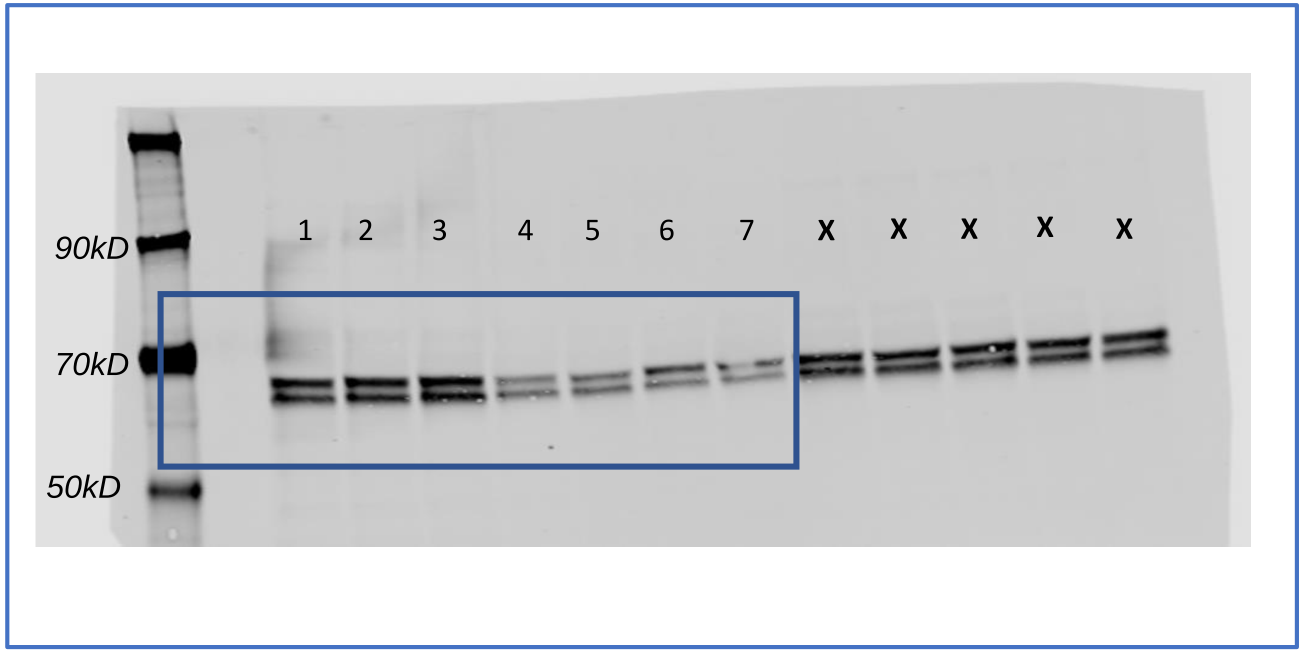


Neurons Transduced with following lentivirus

1. Scrambled shRNA + Okadaic acid 100nM
2. Scrambled shRNA + Okadaic acid 100nM
3. Scrambled shRNA + Okadaic acid 100nM
4. TTBK1 shRNA 1 + Okadaic acid 100nM
5. TTBK1 shRNA 1 + Okadaic acid 100nM
6. TTBK1 shRNA 2 + Okadaic acid 100nM
7. TTBK1 shRNA 2 + Okadaic acid 100nM

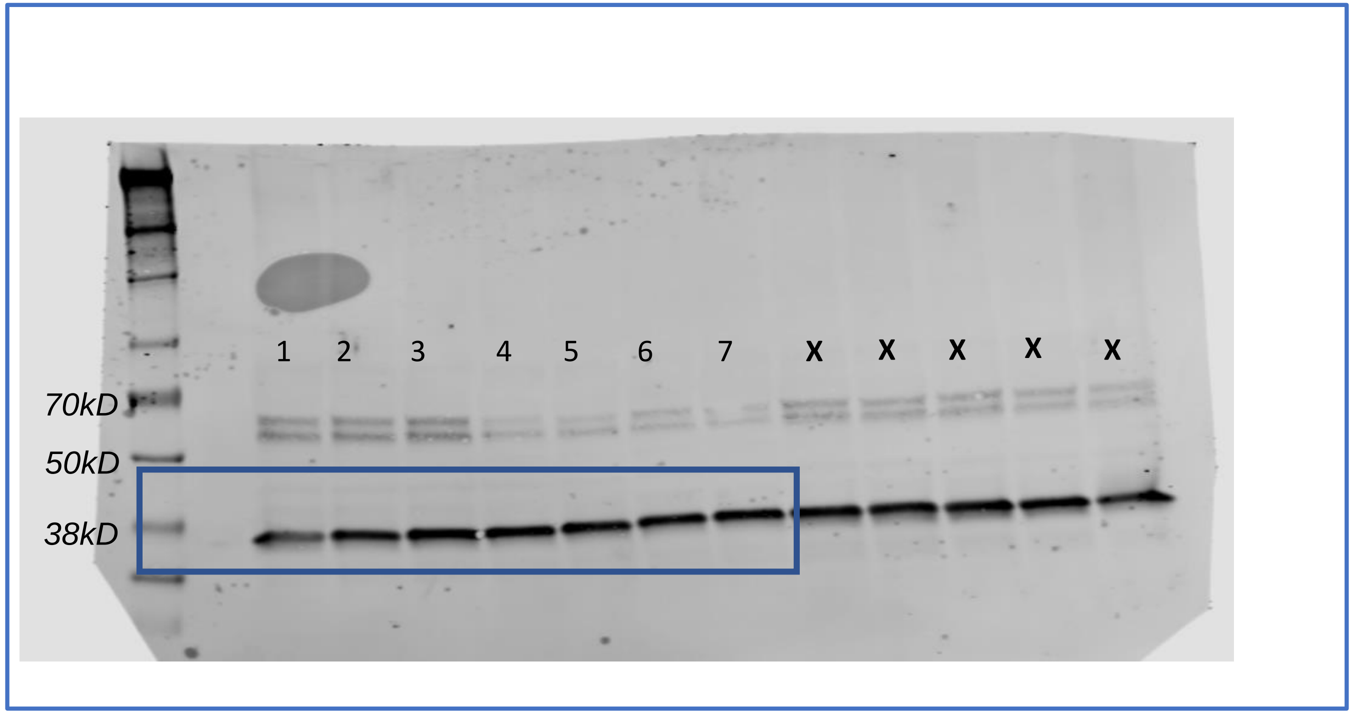


Figure 3B GAPDH

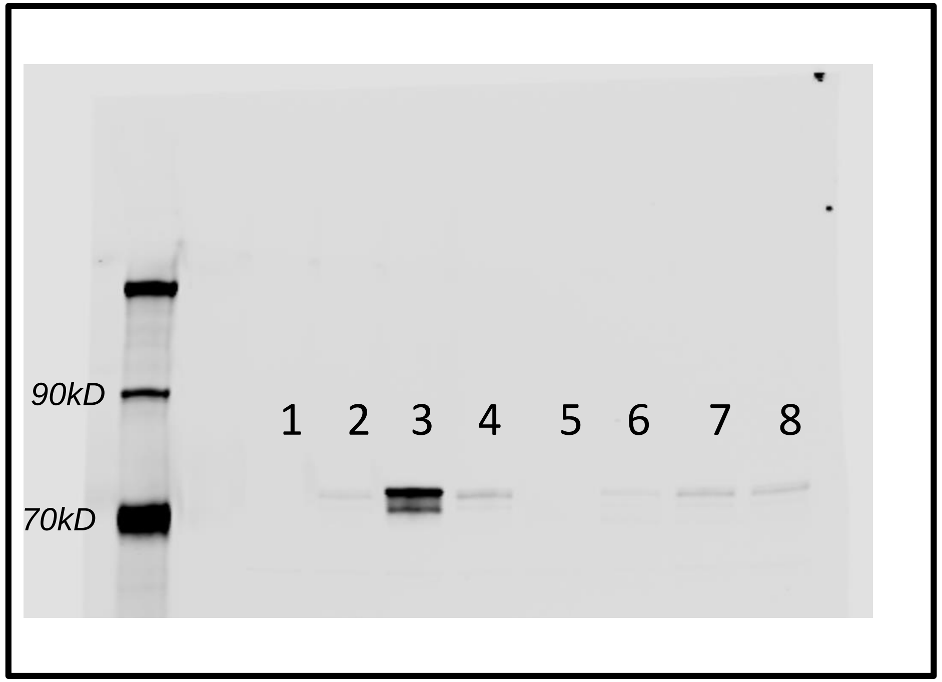


Figure 5C pS422

HEK cells transfected with following plasmids

1) GFP + Empty Plasmid
2) GFP + Tau
3) TTBK1 + Tau
4) TTBK2 + Tau
5) CDK1d + Tau
6) TAOK1 + Tau
7) MAP4K5 + Tau
8) CAMK1a + Tau

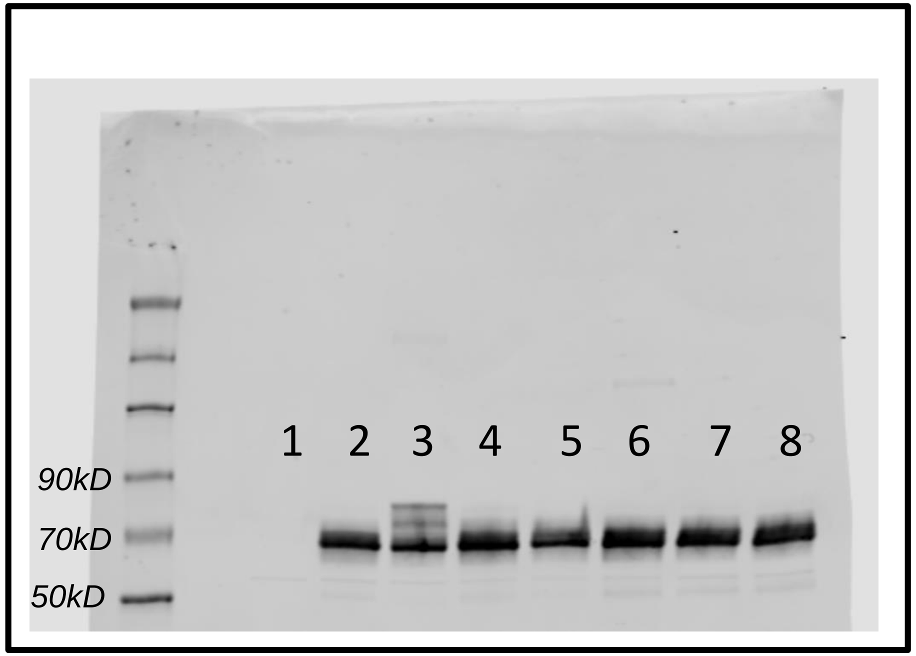


Figure 5C Tau5

HEK cells transfected with following plasmids

1) GFP + Empty Plasmid
2) GFP + Tau
3) TTBK1 + Tau
4) TTBK2 + Tau
5) CDK1d + Tau
6) TAOK1 + Tau
7) MAP4K5 + Tau
8) CAMK1a + Tau

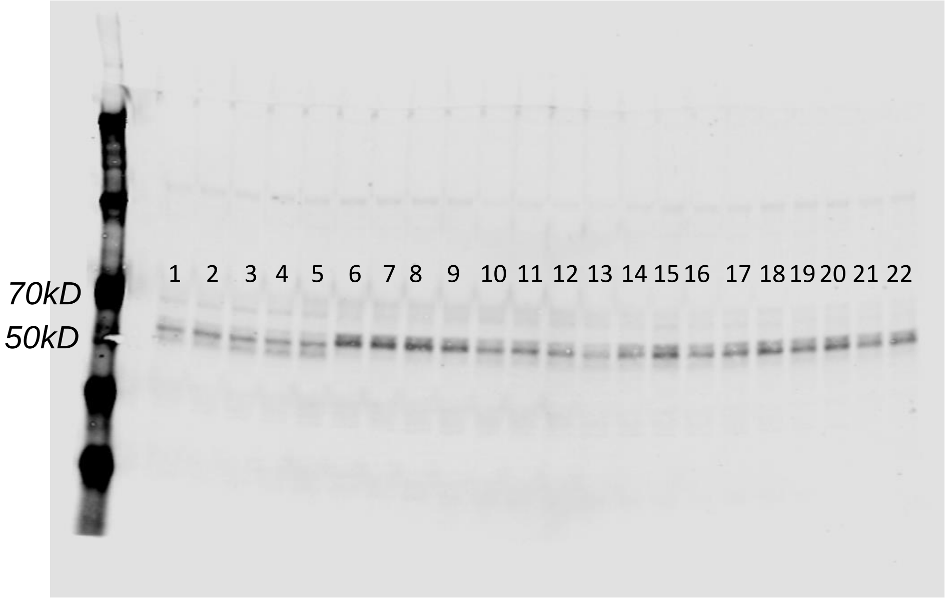


Figure 6A pS422

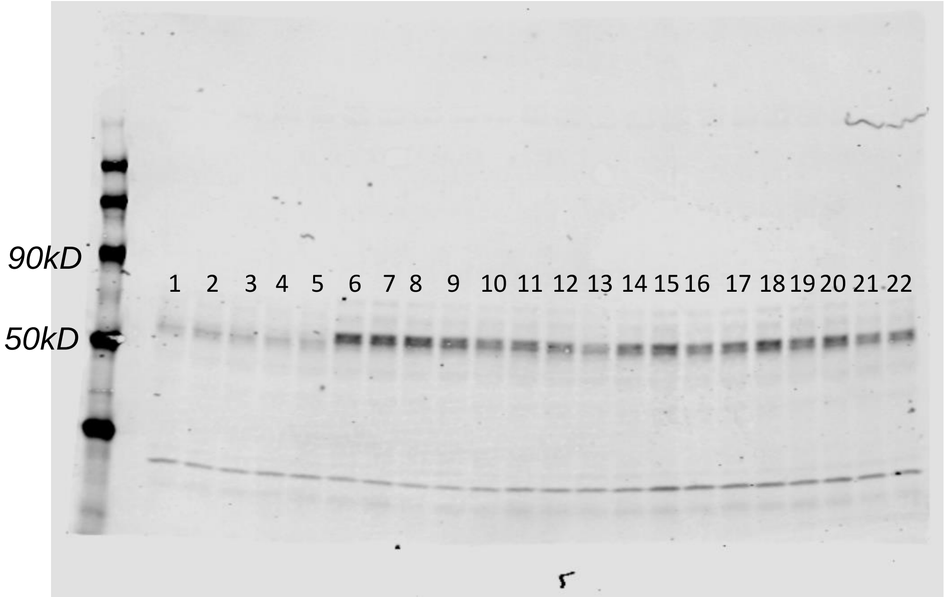


Figure 6A AT180

Mice dosed prior to 1hr of hypothermia

Lanes 1-5: Control - no hypothermia – Vehicle treatment
Lanes 6-9: Hypothermia + Vehicle
Lanes 10-13: Hypothermia + TTBKi 75mg/kg
Lanes 14-17: Hypothermia + TTBKi 50mg/kg
Lanes 18-22: Hypothermia + TTBKi 25mg/kg

For Western blot quantification these lanes were used because these samples were also used in KiNativ proteomics study

- Control = Lanes 1,2,3
- Hypo + Vehicle = Lanes 6,7,8
- Hypo + TTBKi 75mg/kg = Lanes 10,11,12
- Hypo + TTBKi 50mg/kg = Lanes 14, 15, 16
- Hypo + TTBKi 25mg/kg = Lanes 18, 19, 20

All quantifications done on Licor

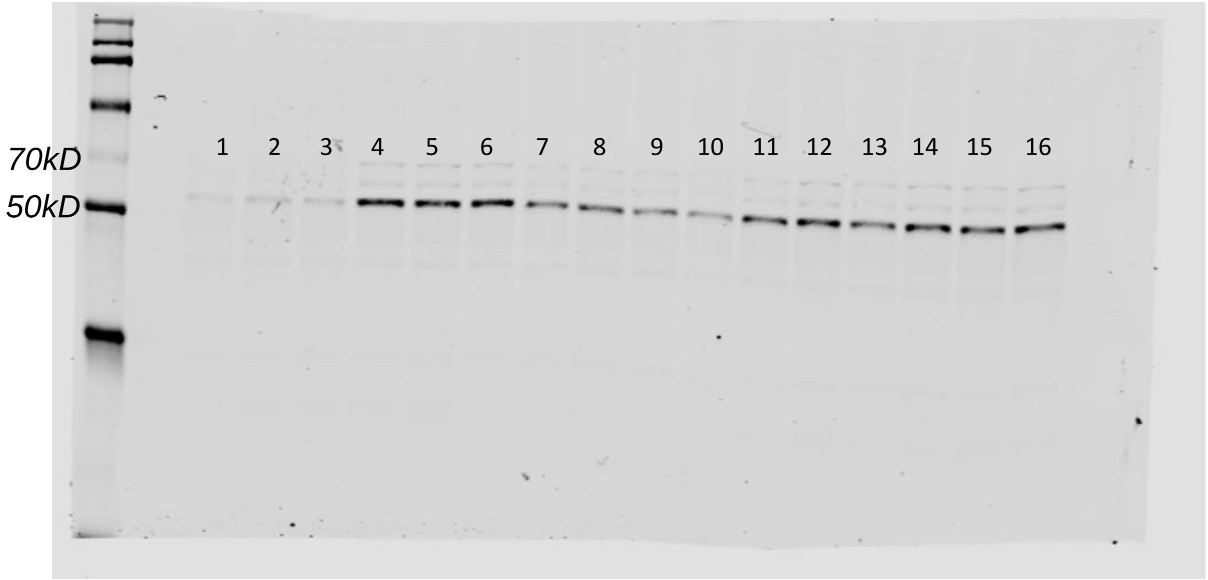


Figure 6A AT8

Mice dosed prior to 1hr of hypothermia

Lanes 1-3: Control - no hypothermia – Vehicle treatment
Lanes 4-6: Hypothermia + Vehicle
Lanes 7-9: Hypothermia + TTBKi 75mg/kg
Lanes 10-12: Hypothermia + TTBKi 50mg/kg
Lanes 13-16: Hypothermia + TTBKi 25mg/kg

All quantifications done on Licor

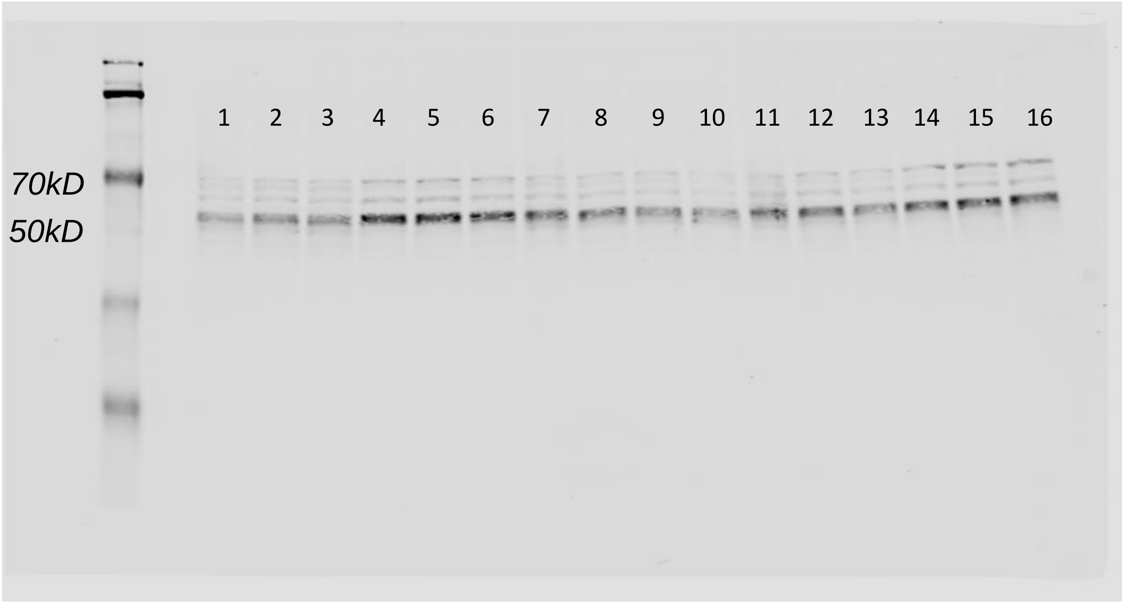


Figure 6A pS396

# Supplemental Figure 1: Western blot demonstrating tau phosphorylation prior to tubulin polymerization assay

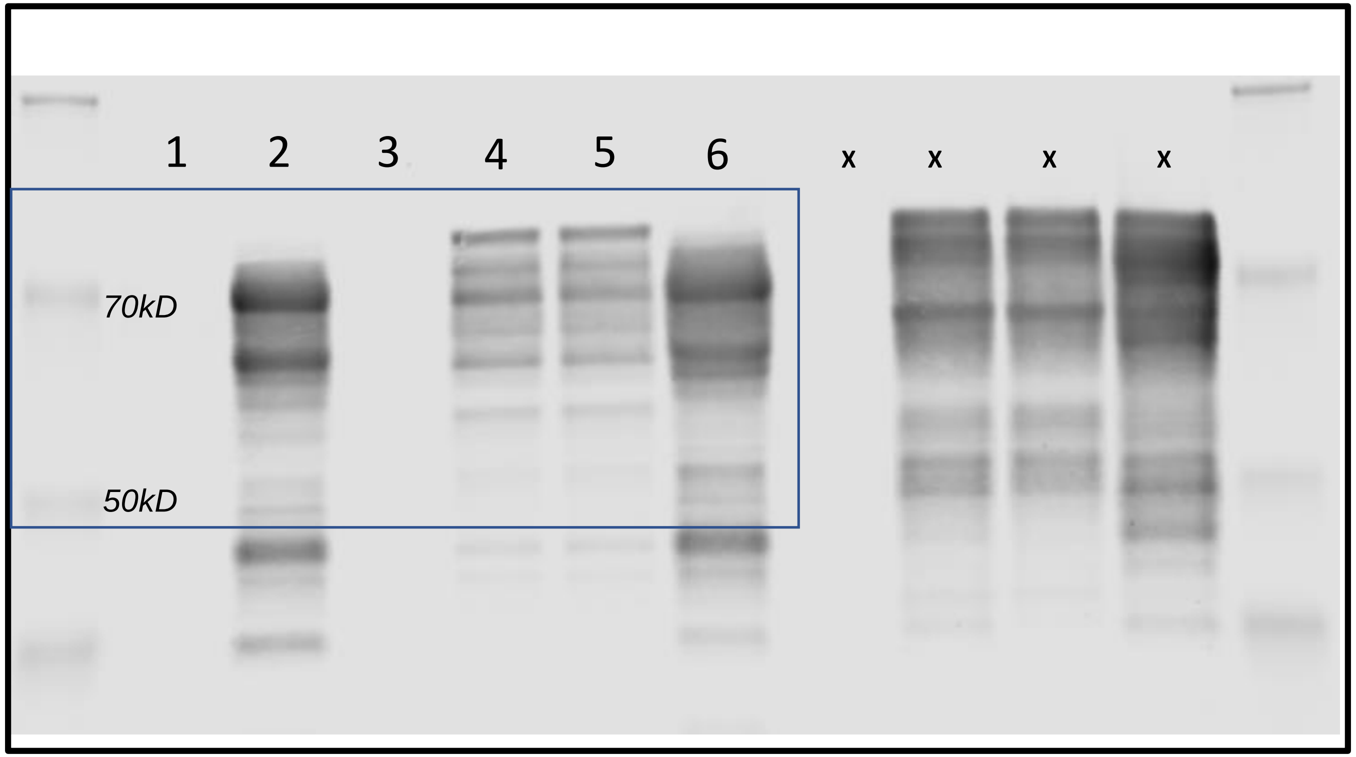


Supp Figure 1 Tau5

HEK cells transfected with following plasmids

1) Empty Plasmid
2) Empty + Tau
3) TTBK1 + Empty
4) TTBK1 + Tau
5) TTBK1 + Tau
6) TTBK1 K63A + Tau

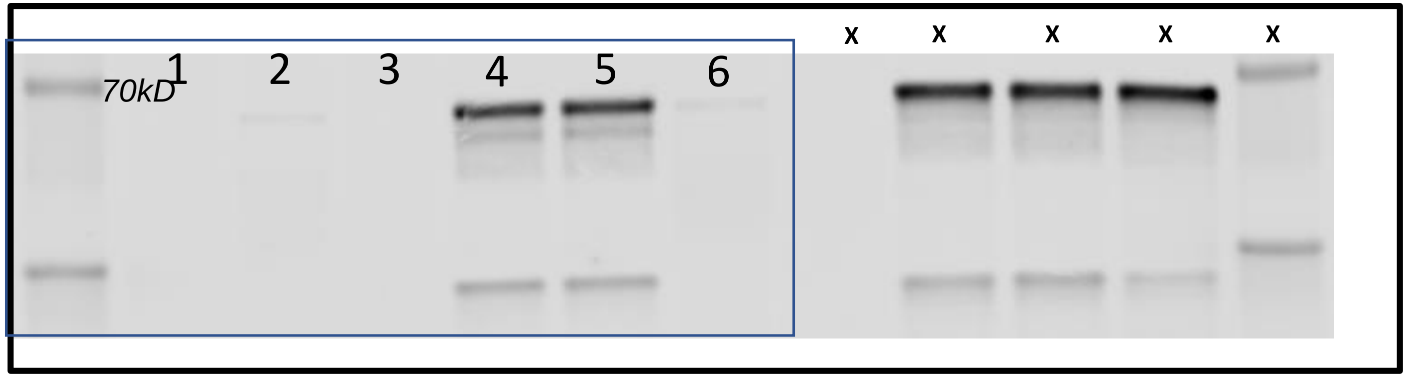


Supp Figure 1 S422

HEK cells transfected with following plasmids

1) Empty Plasmid
2) Empty + Tau
3) TTBK1 + Empty
4) TTBK1 + Tau
5) TTBK1 + Tau
6) TTBK1 K63A + Tau
